# Child and adolescent nutrient intakes from current national dietary surveys of European populations

**DOI:** 10.1017/S0954422418000161

**Published:** 2018-10-31

**Authors:** Holly L. Rippin, Jayne Hutchinson, Jo Jewell, Joao J. Breda, Janet E. Cade

**Affiliations:** 1 Nutritional Epidemiology Group (NEG), School of Food Science and Nutrition, University of Leeds, Leeds LS2 9JT, UK; 2 Division of Noncommunicable Diseases and Promoting Health through the Life-Course, World Health Organization Regional Office for Europe, UN City, Marmorvej 51, 21000 Copenhagen, Denmark

**Keywords:** National diet surveys, WHO European region, Child nutrition, Energy intakes, Macronutrient intakes, Micronutrient intakes, Recommended nutrient intakes

## Abstract

The WHO encourages national diet survey (NDS) implementation to obtain relevant data to inform policies addressing all forms of malnutrition, which remains a pressing issue throughout Europe. This paper provides an up-to-date review on energy, macro- and selected micronutrient intakes in children across WHO Europe using the latest available NDS intakes. It assesses these against WHO recommended nutrient intakes (RNI) to highlight vulnerable groups and areas of concern. Dietary survey information was gathered by Internet searches, contacting survey authors and nutrition experts. Survey characteristics, energy and nutrient intakes were extracted and weighted means calculated and presented by region. Child energy and nutrient intakes were extracted from twenty-one NDS across a third (*n* 18) of the fifty-three WHO Europe countries. Of these, 38 % (*n* 6) reported intakes by socio-economic group, but by various indicators. Energy and macronutrients, where boys and older children had higher intakes, were more widely reported than micronutrients. Most countries met under half of the WHO RNI for nutrients reported in their NDS. Micronutrient attainment was higher than macronutrients, but worst in girls and older children. Only a third, mainly Western, WHO European member states provided published data on child nutrient intakes. Gaps in provision mean that dietary inadequacies may go unidentified, preventing evidence-based policy formation. WHO RNI attainment was poor, particularly in girls and older children. Inconsistent age groups, dietary methodologies, nutrient composition databases and under-reporting hinder inter-country comparisons. Future efforts should encourage countries to conduct NDS in a standardised format by age and sociodemographic variables.

## Introduction

The burden of malnutrition in the form of overweight and obesity, nutrient deficiency and preventable diet-related non-communicable diseases (NCD) is significant and worsening worldwide^(^^1^^,^^2^^)^. In particular, unhealthy diet is one of the four major behavioural risk factors for NCD in all WHO regions^(^^3^^)^, with the European region proportionately suffering the greatest NCD burden. In Europe, the four most common NCD account for 77 % of disease and almost 86 % premature mortality^(^^1^^)^, and overweight and obesity affect a third of children aged 11 years^(^^4^^)^. Childhood obesity has negative health impacts and is associated with educational underachievement, low self-esteem and increased obesity risk in adulthood^(^^5^^)^.

National diet surveys (NDS) have an important role in assessing dietary patterns and intakes in the whole population and informing relevant policy decisions; the WHO European Food & Nutrition Action Plan^(^^1^^)^ explicitly encourages member states to ‘strengthen and expand nationally representative diet and nutrition surveys’. However, NDS provision across Europe is inconsistent. A recent review found that less than two-thirds (thirty-four out of fifty-three) of WHO Europe countries have nationally representative NDS, and that the majority of gaps lie in Central and Eastern European countries (CEEC)^(^^6^^)^. This is concerning, as nutrition policies in these countries may therefore lack an appropriate evidence base.

Novaković *et al.*^(^^7^^)^ examined selected micronutrient intakes in CEEC compared with other European countries and found that CEEC lacked intake data across all ages, particularly in children. The aforementioned recent review by Rippin *et al.*^(^^6^^)^ showed that under a third (seventeen out of fifty-three) of European countries reported energy and nutrient intakes for children aged<18 years from NDS conducted post-2000^(^^6^^)^. This finding is not surprising, as data of this kind are limited. The Global Dietary Database houses information on food and nutrient consumption levels before 2010 in countries globally, but has limited nutrient data, includes some regional rather than national surveys and does not currently cover children^(^^8^^)^. Merten *et al.*^(^^9^^)^ reviewed methodological characteristics and heterogeneity in European NDS, but also included regional child surveys. However, the surveys were limited to European Union member states, only included surveys employing certain dietary assessment methods and did not discuss nutrient intakes.

Despite this lack of data, nutrition and health surveys remain the main source of information on dietary risk factors. For example, a systematic analysis of disease risk in twenty-one regions worldwide between 1990 and 2010 was conducted based on information collated from NDS^(^^10^^)^. Such data are also used to inform policy and identify food and nutrients of most concern. For example, Volatier *et al.*^(^^11^^)^ used NDS to compile a reference list of indicator foods to be used for the validation of nutrient profiling schemes – a policy tool to categorise foods according to their nutritional composition to aid disease prevention and health promotion. These examples demonstrate the importance and range of use that NDS can have in monitoring population diet quality and health, and gathering information on which to base disease–risk prevention policies and address childhood obesity. NDS can help monitor NCD risk factors and malnutrition, identify specific areas of concern, highlight inequalities and evaluate policy impact, thereby ultimately contributing to the promotion of best practice for nutritional health across the region^(^^1^^)^.

A comprehensive, up-to-date review of total nutrient intakes across different European child populations is therefore needed, which could identify where in Europe there is a need to improve diets and whether inequalities exist. In a manner consistent with that published for adults^(^^12^^)^, the present review aims to examine macro- and selected micronutrient intakes in children across the WHO European region via the latest NDS for which nutrient intake data are available, with reference to age-appropriate WHO recommended nutrient intakes (RNI).

## Methods

### Identifying national diet surveys

The methods for identifying and accessing NDS have been reported^(^^6^^)^. Briefly, authors of national surveys within the WHO European region were identified using listed contact names and other information from two main reports of NDS^(^^13^^,^^14^^)^. Where no response was obtained from authors, further general Internet searches were performed on organisations specialising in nutrition to find other potentially useful contact details. Additionally, country responses to WHO Global Nutrition Policy Review 2017 questionnaires were mined to obtain relevant references. Contacts identified were asked to complete a questionnaire to provide information on nationally representative dietary surveys conducted on adults or children at an individual level since 1990, including links or references to relevant reports. For countries without contact details, a systematic database search was performed across Web of Science, MEDLINE and Scopus for nationally representative dietary surveys of adults and children that collected data at an individual level from 1990 to June 2016. Papers returned were screened for relevance according to the criteria in Table 1.

**Table 1:** Survey inclusion and exclusion criteria

Included	Excluded
Surveys conducted at an individual level	Surveys collected at group, i.e. household, level
Nationally representative surveys	Non-nationally representative, regional-only surveys
Results of surveys reported by published and unpublished reports, academic journals and websites	Surveys with data collected before 1990
Surveys that included individuals>2 years	Surveys with samples exclusively<2 years
Surveys based on whole diet rather than specific food groups	Surveys with incomplete food group coverage
	Surveys with small sample sizes (*n*<200)

We found 109^(^^6^^)^ (and have subsequently added recent releases to make 110) nationally representative surveys that collected data on whole diets at an individual level since 1990 across thirty-four of the fifty-three countries in WHO Europe; sixty-nine included children, of which forty-nine were conducted since 2000. Further details of all surveys found are presented in Rippin *et al.*^(^^6^^)^.

### Data extracted

Where available, estimated energy and nutrient intakes by sex and age group were extracted from the latest NDS collected after 2000. For NDS that provided results including and excluding supplements, the latter was used; where not specified, it was assumed that intakes excluded supplements. For children this was extracted from twenty-one surveys from eighteen countries; the Netherlands had two and Ireland three surveys, which covered different child age groups. Mean values were reported in all cases except Dutch children aged 7–8 years, which used medians – these were extracted and used instead. The eighteen countries were grouped into regions – Western, Northern and Central and Eastern Europe. For some countries (France, Latvia, the Netherlands and Spain), more recent surveys had been conducted, but intake data were not yet available. Energy intakes reported in kcal were converted to MJ for consistency across studies. Appendix 1 and Appendix 2 list the availability of selected nutrients reported from the latest surveys collected after 2000.

All macronutrients reported by the twenty-one surveys were included in the data extraction (see Table 2), but micronutrients extracted (see Table 3) were limited to those explicitly mentioned in the WHO European Food and Nutrition Action Plan^(^^1^^)^ as being currently important to population health in the region. See Appendix 1 and Appendix 2 for all nutrient intakes extracted.

**Table 2:** Macronutrients of interest in dietary surveys and corresponding WHO recommended nutrient intake (RNI)

	WHO RNI format	Lower RNI	Upper RNI	Single value
Energy (MJ and kcal)	N/A	N/A	N/A	N/A
Carbohydrates (g and %E)	Target	55 %E	75 %E	
Sugars (g)	N/A	N/A	N/A	N/A
Sucrose (g)	Maximum	5 %E	10 %E	
Fibre (g)	Target			25 g
Total fat (g)	Maximum	15 %E	30 %E	
Saturated fats (g)	Maximum			10 %E
MUFA (g)	N/A	N/A	N/A	N/A
PUFA (g)	Target	6 %E	10 %E	
TFA (g)	Maximum			<1 %E
Protein (g)	Target	10 %E	15 %E	
*n-*3 Fatty acids (g)	Target	1 %E	2 %E	
*n*-6 Fatty acids (g)	Target	5 %E	8 %E	

N/A, not applicable; %E, percentage energy; TFA, *trans*-fatty acids.

**Table 3:** Micronutrients of interest in dietary surveys and corresponding WHO recommended nutrient intake (RNI)

	RNI format	1–3 years	4–6 years	7–9 years	10–18 years
Total folate (µg)	Minimum	100 µg	200 µg	300 µg	400 µg
Vitamin B_12_ (µg)	Minimum	0·9 µg	1·2 µg	1·8 µg	2·4 µg
Vitamin D (µg)	Target	5 µg	5 µg	5 µg	5 µg
Ca (mg)	Minimum	500 mg	600 mg	700 mg	1300 mg
K (mg)*	Minimum/target	800 mg	1100 mg	2000 mg	3100–3500 mg
Na (mg)*	Maximum	500 mg	700 mg	1200 mg	1600 mg
Fe (mg)*	Minimum	6·9 mg	6·1 mg	8·7 mg	11·3–14·8 mg
Iodine (µg)	Minimum	90 mg	90 mg	120 mg	150 mg
Zn (mg)	Minimum	4·1 mg	4·8 mg	5·6 mg	7·2–8·6 mg

* RNI are derived from the WHO except Fe, K and Na, where UK RNI have been used instead, as WHO Fe RNI values are based on different bioavailabilities and K and Na values are downweighted based on energy requirements for children relative to adults.

WHO RNI were used to assess intake adequacy in the population majority and highlight areas of concern in the absence of raw NDS data from sufficient countries to determine the prevalence of inadequacy in relation to the percentage of the population below the estimated average requirements. The exception was energy, where RNI changed in yearly increments, so were not sufficiently compatible with survey age groupings^(^^15^^–^^20^^)^. Additionally, WHO RNI for Fe are given for different bioavailabilities, so UK Reference Nutrient Intakes (RNI) were used instead^(^^21^^)^. UK RNI were also used for K and Na, as WHO RNI recommend downweighting values based on energy requirements for children relative to adults. The RNI for MUFA is calculated by the difference between total fat and the sum of SFA, PUFA and *trans*-fatty acids (TFA), so has not been included. The WHO RNI for free sugars^(^^19^^)^ has been adopted as the RNI for added sugars, as no WHO RNI exists for added sugars, yet the majority of surveys that reported sugars used the added rather than free sugar definition. The added sugars definition is similar but more restrictive to that of free sugars, meaning that free sugar intake would not be overestimated. Depending on the nutrient, RNI were variously maximum, minimum or target amounts (see Tables 2 and 3).

Energy and selected nutrient intakes reported by age group and sex in these latest surveys collected after 2000 were graphed. To harmonise the data where possible, units of measurement were converted to common standard units. Omega-3 (*n*-3) and omega-6 (*n*-6) fatty acids were reported variously in surveys, including *n*-3, *n*-6, linoleic acid and α-linolenic acid in g/d and percentage energy (%E) and EPA+DHA in mg/d. These were converted to g and %E and grouped into *n*-3 and *n*-6 fatty acids for clarity. ‘Added sugars’ is used as a proxy term for sucrose, as the countries reporting this nutrient typically referred to it as ‘added sugars’ or did not specify.

Additionally, estimated mean intakes by sex for two age groups split roughly by those aged<10 years and≥10 years (to 18 years) were determined for each country, and also for European regions and Europe overall. This cut-off was chosen because 10 years was a common boundary for RNI split by age. Age ranges for reported and extracted means that spanned the 10-year cut-off contained a larger proportion of≥10-year-olds in all cases, so were allocated to that group. This occurred in seven countries (Cyprus, Ireland, Latvia, the Netherlands, Spain, Turkey and the UK), where only Latvia included children aged<9 years (7–16 years). The UK 4- to 10-year age group was included in the<10 years group. Some countries did not separate by sex in the youngest ages – in these instances the same mean intake was used for both girls and boys. Where mean intakes were reported by a country for more than one age group<10 years, or more than one age group≥10 years, the numbers of children/adolescents surveyed in the NDS in each age group were used to weight the reported means to produce estimated mean intakes for those aged<10 years and≥10 years. For instance, mean intakes for Belgium were reported and extracted for 3- to 5-year-olds, 6- to 9-year-olds, 10- to 13-year-olds and 14- to 17-year-olds; the mean intake reported for boys aged 3–5 years was multiplied by the number of boys surveyed for that age group, and added to a similar calculation for the 6- to 9-year-olds; the sum of these was then divided by the total number of boys aged<10 years to produce an estimated mean for<10 years for Belgium. Where countries had multiple NDS (Ireland, the Netherlands), age ranges ran concurrently rather than overlapping, so the NDS were grouped and used to estimate the mean intakes for those aged<10 years and≥10 years as described above. The mean intakes for each European region and Europe overall were estimated by multiplying the<10 years or≥10 years means for each country and sex by the national population aged<19 years for each country^(^^22^^–^^24^^)^. The resulting value for each country was summed and then divided by the total sum of the national child populations in each European region, then Europe as a whole. The same population values were used for both the<10 years and≥10 years groups, assuming similar population ratios. These population weightings made the estimated means roughly generalisable to the European regions and Europe as a whole.

Characteristics of the twenty-one surveys were also extracted and tabled; these were: country name, survey name, year of survey (data collection), source, sample size, age range, dietary methodology and the nutrient reference database underpinning the survey. The number and percentage of WHO RNI not met were recorded for the nutrients and sex/age groups for which they were reported. Where reported, surveys presenting nutrient intakes by socio-economic group based on social class, income (continuous or grouped), occupation and education level were also noted.

## Results

### Data extracted

The scope of NDS coverage across Europe has previously been documented^(^^6^^)^. Energy and nutrient intakes (excluding supplements) for children aged ≤18 years were extracted from twenty-one surveys across eighteen countries from three regions: two of five Northern European countries (Denmark, Norway); ten of seventeen Western European countries (Austria, Belgium, France, Germany, Ireland, Italy, The Netherlands, Portugal, Spain, UK) and six of thirty-one CEEC (Bulgaria, Cyprus, Estonia, Latvia, Slovenia, Turkey). Table 4 shows the characteristics of these surveys. Child energy and nutrient intakes could not be extracted for 66 % (thirty-five) of European countries for various reasons, from lack of availability to incompatible age-group structure. Nineteen of these countries, mainly CEEC, had no identifiable nationally representative survey, making up over a third of WHO Europe countries. The Andorran NDS surveyed children, but the lowest age group (12–24 years) spanned adults and children, so intake data were not included in the results or graphs.

**Table 4:** National diet surveys across countries in WHO Europe 2000–2016 with reported nutrient intakes for children and adolescents

Country	Survey name	Survey year	Source*	Sample size	Sample age (years)	Dietary methodology	Nutrient reference database	Nutrient intakes by SEG, Y/N†	WHO RNI not met‡	Reference
Austria	Austrian Nutrition Report 2012 (OSES)	2010–2012	2	1002	7–14; 18–80	3 d diary (consecutive) (children); 2×24 h recall (adults). Face-to-face and telephone interview	Analysis run with software ‘(nut.s) science’ based on Bundeslebensmittelschlüssel 3·01/Goldberg cut-offs for data cleaning	N	69 % (75/108)	(54)
Belgium	Belgium National Food Consumption Survey (BNFCS) 2014	2014–2015	1 and 2	3146	3–64	2×24 h recall. Face-to-face electronic interview	The NIMS Belgian Table of Food Composition (Nubel); Dutch Food Composition Database (NEVO)	N	68 % (73/108)	(55,56)
Bulgaria	National Survey on Nutrition of Infants and Children Under 5 and Family Child Rearing 2007	2007	2	1723	0–5	2×24 h recall via mother (non-consecutive). Face-to-face interview with the mother	FCTBL_BG (Food Composition Tables – Bulgaria)	N	60 % (30/50)	(57–59)
Cyprus	A study of the dietary intake of Cypriot children and adolescents aged 6–18 years	2009–2010	2	1414	6–18	3 d food record (consecutive including one weekend). Self-completed	USDA Nutrient Database for Standard Reference and Research	N	75 % (45/60)	(60)
Denmark	Danish National Survey of Diet and Physical Activity (DANSDA) 2011–2013	2011–2013	2	3946	4–75	7 d diary (consecutive). Self-completed (by mother/carer 4–15 years)	Danish Food Composition Databank	N	60 % (41/68)	(61)
Estonia	National Dietary Survey	2014–2015	1	4906	4 months–74 years	2×24 h recall (age≥10 years); 2×24 h food diary (age<10 years); FFQ (age >2 years). Face-to-face electronic interview		Y – income, poverty threshold, education	64 % (84/132)	(62,63)
France	Individual National Food Consumption Survey (INCA3)	2014–2015	2	5855	0–79	3×24 h recalls (15+ years); 3 d diary (0–14 years). Non-consecutive including weekend; telephone interview	Food Composition Database CIQUAL of Anses	Y – education, parent occupational category	82 % (56/68)	(64)
Germany	German National Nutrition Survey (Nationale Verzehrstudie) II (NVSII)	2005–2007	1 and 3	15 371	14–80	DISHES diet history interview, 24 h recall, diet weighing diary (2×4 d). Face-to-face electronic interview	Bundeslebensmittelschlüssel (BLS)	N	54 % (14/26)	(65,66)
Ireland	National Pre-school Nutrition Survey	2010–2011	1	500	1–4	4 d weighed food diary (consecutive). Self-completed (by carer)	McCance and Widdowson’s The Composition of Foods, 5th and 6th editions	Y – social class and education	57 % (110/192)	(67,68)
	National Teens’ Food Survey	2005–2006	1	441	13–17	7 d semi-weighed food diary (consecutive). Self-completed	McCance and Widdowson’s The Composition of Foods, 5th and 6th editions	Y – social class and education		(69–71)
	National Children’s Food Survey	2003–2004	1	594	5–12	7 d weighed food diary (consecutive). Self-completed	McCance and Widdowson’s The Composition of Foods, 5th and 6th editions	Y – social class and education		(70–72)
Italy	Third Italian National Food Consumption Survey INRAN-SCAI 2005–2006	2005–2006	2	3323	0·1–97·7	3 d diary (consecutive). Self-completed	Banca Dati di Composizione degli Alimenti	N	65 % (42/72)	(73)
Latvia	Latvian National Food Consumption Survey 2007–2009	2008	1	1949	7–64	2×24 h recall, FFQ. Face-to-face interview	Latvian National Food Composition Data Base 2009	N	100 % (2/2)	(74)
Netherlands	Dutch National Food Consumption Survey 2007–2010 (DNFCS 2007–2010)	2007–2010	1 and 2	3819	7–69	2×24 h recalls. Telephone (adults)/face-to-face (children) interview	Dutch Food Composition Database (NEVO)	Y – education	51 % (75/148)	(75–77)
	Dutch National Food Consumption Survey – young children (DNFCS 2008)	2005–2006	1	1279	2–6	2 d diary (non-consecutive). Self-completed (by adult)	Dutch Food Composition Database (NEVO)	N		(78)
Norway	UNGKOST 3	2015–2016	1	1721	4–13	4 d online diary plus FFQ (consecutive). Self-completed via web	Norwegian Food Composition Tables	Y – parental education	70 % (59/84)	(79,80)
Portugal	National Food and Physical Activity Survey (IAN-AF)	2015–2016	4	4221	3 months–84 years	2×24 h recall (non-consecutive) and FPQ (electronic interview). 2 d food diary for children<10 years. Face-to-face electronic interview	Portuguese Food Composition Table (INSA)	N	61 % (39/64)	(81,82)
Slovenia	Dietary Intake of Macro- and Micronutrients in Slovenian Adolescents	2012	2	2224	15–16	FFQ	German Bundeslebensmittelschlüssel (BLS) 3·02	N	44 % (15/34)	(83)
Spain	ANIBES Study	2013	2	2285	9–75	3 d diary + 24 h recall (consecutive). Face-to-face, telephone (interview), tablet and camera (self-report)	Tablas de Composición de Alimentos, 15th edition	N	67 % (16/24)	(84–86)
Turkey	Turkey Nutrition and Health Survey 2010 (TNHS)	2010	2	14 248	0–100	24 h recall, FFQ. Face-to-face interview	BEBS Nutritional Information System Software; Turkish Food Composition Database	N	68 % (116/170)	(87,88)
UK	National Diet and Nutrition Survey Rolling Programme (NDNS RP 2008–2012)	2008–2012	2	6828	1·5–94	4 d diary (consecutive). Self-completed (by carer 1·5–11 years)	McCance and Widdowson’s The Composition of Foods integrated dataset	Y – income	74 % (80/108)	(89)

SEG, socio-economic group; Y, yes; N, no; RNI, recommended nutrient intake; USDA, United States Department of Agriculture; FPQ, Food preference questionnaire.

* 1=email contacts; 2=general Internet searches; 3=Micha *et al.*^(^^14^^)^; 4=WHO Global Nutrition Policy Review 2017 extracted information.

† Countries that have reported nutrient intakes by SEG in addition to age and sex.

‡ The right values in parentheses provide the number of RNI not met by each age/sex group out of a total number of RNI for age/sex group for each nutrient reported by that country. The left value is this as a percentage.

All twenty-one NDS that reported nutrient intakes included energy; however, Latvia reported no other macronutrients. The majority (*n* 20) reported protein, carbohydrate and fat intakes and most reported fibre intakes (*n* 19) (see Table 5). Most NDS included intake data on saturated fats (*n* 19), and MUFA and PUFA (*n* 18). However, less than half (*n* 7) NDS included TFA intakes. Most NDS (*n* 16) included either total or added sugars/sucrose; however, six NDS included neither. Just over half the countries included either *n*-3 (*n* 7) or *n*-6 (*n* 7) fatty acid intakes in some form; six NDS included both.

**Table 5:** Estimated means for<10 years and≥10 years by country and region for macronutrients in twenty-one national dietary surveys in the WHO Europe region*

Country	Energy (MJ)	Protein (g)	CHO (g)	Sugars (g)	Sucrose (g)	Fibre (g)	Total fat (g)	Saturated fats (g)	MUFA (g)	PUFA (g)	TFA (g)	*n*-3 (g)	*n*-6 (g)
Bulgaria (1–4 years)	National Survey on Nutrition of Infants and Children Under 5 Years and Family Child Rearing 2007												
Girls<10 years	5·8	47	175	31		13·1	59	15	11	9·4			
Boys<10 years	6·1	49	175	31		13·1	59	15	11	9·4			
Girls≥10 years													
Boys≥10 years													
Cyprus (6–8·9 years; 9–18·9 years)	A study of the dietary intake of Cypriot children and adolescents aged 6–18 years 2009–2010												
Girls<10 years	7·6	73	223			14·6	69	28	30	9·7			
Boys<10 years	7·8	75	226			14·8	72	29	31	10·3			
Girls≥10 years	7·5	73	207			14·1	73	28	33	10·6			
Boys≥10 years	8·5	88	225			14·9	85	33	38	12·7			
Estonia (2–9 years; 10–17 years)	National Dietary Survey 2014–2015												
Girls<10 years					56			27	21	8·7	0·5	1·3	6·7
Boys<10 years					61			30	24	9·9	0·6	1·4	7·6
Girls≥10 years	6·6	55	205		52	14·3	62	26	21	9·9	0·4	1·4	7·8
Boys≥10 years	8·8	78	269		63	18·2	83	34	29	13·7	0·6	2·2	10·8
Latvia (7–16 years)	Latvian National Food Consumption Survey 2007–2009												
Girls<10 years													
Boys<10 years													
Girls≥10 years	6·9												
Boys≥10 years	8·2												
Slovenia (15–16 years)	Dietary Intake of Macro- and Micronutrients in Slovenian Adolescents 2012												
Girls<10 years													
Boys<10 years													
Girls≥10 years	9·7	86	379	195	110	31	82	35	29	17·0			
Boys≥10 years	12·7	96	370	170	103	28	82	36	30	16·0			
Turkey (2–8 years; 9–18 years)	Turkey Nutrition and Health Survey 2010 (TNHS)												
Girls<10 years	5·3	38	158			12·5	51	17	17	13·4		1·0	12·4
Boys<10 years	5·5	41	163			12·9	54	19	18	14·3		1·0	13·3
Girls≥10 years	7·1	50	220			18·7	66	21	22	17·9		1·2	16·6
Boys≥10 years	8·3	61	257			20·6	76	26	25	19·8		1·4	18·3
CEEC mean girls<10 years	5·3	39	159	31	56	13	52	17	16	13	0·5	1·0	12·3
CEEC mean boys<10 years	5·6	42	164	31	61	13	55	19	17	14	0·6	1·0	13·2
CEEC mean girls≥10 years	7·1	51	222	195	87	19	66	22	22	18	0·4	1·2	16·5
CEEC mean boys≥10 years	8·4	62	258	170	87	21	76	26	26	20	0·6	1·4	18·2
Denmark (4–9 years; 10–17 years)	Danish National Survey of Diet and Physical Activity (DANSDA) 2011–2013												
Girls<10 years	7·7	64	220		46	19·0	73	29	26	11·0	1·2		
Boys<10 years	8·5	71	243		50	21·0	80	32	28	13·0	1·3		
Girls≥10 years	7·8	67	222		53	17·0	73	30	26	11·0	1·1		
Boys≥10 years	9·9	90	277		67	20·0	94	38	34	14·0	1·4		
Norway (4–9 years; 13 years)	UNGKOST 3 2015–2016												
Girls<10 years	6·4	59	189		43	14·6	56	24	19	8·6			
Boys<10 years	7·1	67	207		44	16·6	62	26	21	9·2			
Girls≥10 years	7·4	68	219		60	15·0	66	27	23	10·0			
Boys≥10 years	8·6	83	247		64	17·0	76	31	27	11·0			
North mean girls<10 years	7·1	61	205		44	17	65	26	23	10	1·2		
North mean boys<10 years	7·8	69	225		47	19	71	29	25	11	1·3		
North mean girls≥10 years	7·6	67	221		56	16	70	29	25	11	1·1		
North mean boys≥10 years	9·3	87	262		66	19	85	35	31	13	1·4		
Austria (7–9 years, 10–14 years)	Austrian Nutrition Report (OSES) 2010–2012												
Girls<10 years	8·0	62	248		53	17·0	72	32	23	12·7		1·5	10·0
Boys<10 years	8·0	62	245		58	18·0	73	34	22	10·7		1·3	9·6
Girls≥10 years	7·3	62	222		48	16·1	66	30	22	10·3		1·2	9·3
Boys≥10 years	8·2	69	247		49	17·6	75	31	24	13·5		1·4	11·2
Belgium (3–9 years; 10–17 years)	Belgian National Food Consumption Survey (BNFCS) 2014–2015												
Girls<10 years	6·2	51	186	99		13·6	57	23	21	9·6	0·7		
Boys<10 years	6·8	56	205	111		13·4	62	25	22	9·6	0·7		
Girls≥10 years	7·8	64	221	107		15·5	74	27	27	12·4	0·7		
Boys≥10 years	9·4	79	270	133		16·4	88	32	32	15·0	0·9		
France (0–10 years; 11–17 years)	Individual National Food Consumption Survey (INCA3) 2014–2015												
Girls<10 years	6·0	53	176	95		12·7	54	24	18	6·4		0·9	4·7
Boys<10 years	6·6	58	199	103		13·8	57	26	19	6·6		0·8	5·0
Girls≥10 years	7·6	70	226	98		16·1	66	28	22	8·5		1·0	6·2
Boys≥10 years	8·9	83	262	111		18·1	77	33	26	9·9		1·1	7·3
Germany (14–18 years)	German National Nutrition Survey (Nationale Verzehrstudie) II (NVSII) 2005–2007												
Girls<10 years													
Boys<10 years													
Girls≥10 years	8·8	66	274			23·2	77						
Boys≥10 years	12·1	94	355			26·0	110						
Ireland (1–8 years; 9–17 years)	National Pre-School Nutrition Survey 2010–2011; National Children’s Nutrition Survey 2003–2004; National Teens Nutrition Survey 2005–2006												
Girls<10 years	5·4	46	171	76	37	10·5	48	21	16	6·5		0·6	
Boys<10 years	5·5	47	177	76	37	10·8	48	22	17	6·5		0·6	
Girls≥10 years	7·1	58	224			9·7	66	27	23	10·5			
Boys≥10 years	8·9	77	281			12·2	82	34	29	12·5			
Italy (0–9·9 years; 10–17·9 years)	Third Italian National Food Consumption Survey INRAN-SCAI 2005–2006												
Girls<10 years	7·3	67	220	83		13·1	72	24	33	8·7			
Boys<10 years	7·3	67	220	83		13·1	72	24	33	8·7			
Girls≥10 years	8·7	82	263	88		16·4	86	27	40	11·1			
Boys≥10 years	10·8	99	327	108		18·1	105	33	49	13·7			
Netherlands (2–8 years; 9–18 years)	Dutch National Food Consumption Survey – young children (DNFCS) 2008; Dutch National Food Consumption Survey (DNFCS) 2007–2010												
Girls<10 years	6·3	48	209	127		13·0	53	20		14·0	1·2		
Boys<10 years	6·6	50	218	132		14·0	54	21		14·0	1·3		
Girls≥10 years	8·5	67	257	134		16·7	76	29	27	14·2	1·2	1·4	11·6
Boys≥10 years	10·7	81	312	159		19·6	94	35	34	18·1	1·5	1·7	15·0
Portugal (0–9 years; 10–17 years)	National Food and Physical Activity Survey (IAN-AF) 2015–2016												
Girls<10 years	5·7	58	175	89		12·8	46	21	21	7·2	0·7		5·9
Boys<10 years	5·9	56	180	90		13·2	47	21	21	7·4	0·7		6·9
Girls≥10 years	7·9	83	219	88		16·1	67	29	27	11·0	1·2		10·5
Boys≥10 years	9·7	104	273	107		18·2	81	35	32	13·1	1·5		13·1
Spain (9–17 years)	ANIBES 2013												
Girls<10 years													
Boys<10 years													
Girls≥10 years	7·8	72	208	88		11·7	80	26	32	13·7			
Boys≥10 years	8·7	83	227	92		11·8	89	30	37	14·8			
UK (1·5–10 years; 11–18 years)	National Diet and Nutrition Survey Rolling Programme (NDNS RP) years 1–4 2008–2012												
Girls<10 years	5·8	50	187	88		9·9	52	21	18	7·7	1·0	1·2	6·6
Boys<10 years	6·0	52	198	92		10·5	54	22	19	8·0	1·0	1·2	6·8
Girls≥10 years	6·6	56	211	90		10·7	60	22	23	10·1	1·1	1·6	8·5
Boys≥10 years	8·3	74	265	116		12·8	74	28	28	11·9	1·4	1·9	10·0
West mean girls<10 years	6·3	55	194	92	46	12	57	23	22	8	1·0	1·0	5·9
West mean boys<10 years	6·6	57	205	97	49	13	59	24	23	8	1·0	1·0	6·1
West mean girls≥10 years	7·9	68	237	95	48	16	72	26	28	11	1·1	1·3	8·0
West mean boys≥10 years	9·7	86	289	113	49	18	90	31	33	13	1·4	1·5	9·6
Europe mean girls<10 years	6·0	50	183	91	46	12	56	21	20	10	1·0	1·0	8·6
Europe mean boys<10 years	6·3	53	192	95	48	13	58	22	21	10	1·0	1·0	9·2
Europe mean girls≥10 years	7·7	64	233	95	58	17	71	25	26	13	1·1	1·3	11·4
Europe mean boys≥10 years	9·4	80	281	113	63	18	86	30	31	15	1·4	1·5	13·1

CHO, carbohydrates; TFA, *trans*-fatty acids; CEEC, Central and Eastern European countries.

* Where mean intakes were reported by a country for more than one age group<10 years, or more than one age group≥10 years, the numbers of children/adolescents surveyed in the national diet survey for each age group and sex were used to weight the reported means to produce estimate mean intakes for those aged<10 years and those aged≥10 years for each nutrient. Countries that span the 10-year boundary are: Cyprus (9–13·9 years); Ireland (9–12 years); Latvia (7–16 years); the Netherlands (9–13 years); Spain (9–12 years); Turkey (9–11 years) and the UK (4–10 years). For each nutrient regional weighted means for North, West and Central and Eastern Europe and Europe overall were calculated by weighting the<10 years and≥10 years country means shown in the table by the total child population in that country^(^^22^^–^^24^^)^.

Micronutrients were less widely covered by the twenty-one surveys – Spain reported no micronutrient intakes and Latvia only included Na (see Table 6). Ca and Fe were reported by all but two surveys (Latvia and Spain did not), whilst vitamins B_12_ and D were reported by all but three (Latvia, Spain and Cyprus did not). Iodine was the least reported micronutrient, by just over half (*n* 11) of the surveys.

**Table 6:** Estimated means for<10 years and≥10 years by country and region for micronutrients in twenty-one national dietary surveys in the WHO Europe region*

Survey	Total folate (µg)	Vitamin B_12_ (µg)	Vitamin D (µg)	Ca (mg)	K (mg)	Na (mg)	Fe (mg)	Iodine (µg)	Zn (mg)
Bulgaria (1–4 years)	National Survey on Nutrition of Infants and Children Under 5 and Family Child Rearing 2007								
Girls<10 years	117	2·3	2·1	541		1637	5·7		5·9
Boys<10 years	117	2·3	2·1	541		1639	5·7		5·9
Girls≥10 years									
Boys≥10 years									
Cyprus (6–8·9 years; 9–18·9 years)	A study of the dietary intake of Cypriot children and adolescents aged 6–18 years 2009–2010								
Girls<10 years				930	2311	2283	10·9		
Boys<10 years				957	2337	2331	11·4		
Girls≥10 years				866	2161	2292	10·5		
Boys≥10 years				974	2432	2699	12·2		
Estonia (2–9 years; 10–17 years)	National Dietary Survey 2014–2015								
Girls<10 years	142	3·8	2·0	671	2449	1147	8·1	108	6·7
Boys<10 years	150	4·7	2·4	738	2689	1314	9·1	122	7·6
Girls≥10 years	156	4·4	2·2	666	2657	1448	9·6	109	7·2
Boys≥10 years	191	5·5	3·3	888	3421	2085	12·4	150	10·2
Latvia (7–16 years)	Latvian National Food Consumption Survey 2007–2009								
Girls<10 years									
Boys<10 years									
Girls≥10 years						2000			
Boys≥10 years						2840			
Slovenia (15–16 years)	Dietary Intake of Macro- and Micronutrients in Slovenian Adolescents 2012								
Girls<10 years									
Boys<10 years									
Girls≥10 years	276	5·9	4·0	1176	3770	4191	16·0	205	12·4
Boys≥10 years	255	6·7	4·0	1094	3494	4059	16·0	181	13·5
Turkey (2–8 years; 9–18 years)	Turkey Nutrition and Health Survey 2010 (TNHS)								
Girls<10 years	200	2·1	0·9	520	1665	1048	7·0	45	5·9
Boys<10 years	205	2·4	1·1	550	1729	1114	7·4	48	6·4
Girls≥10 years	282	3·6	0·9	553	2065	1591	9·6	53	8·0
Boys≥10 years	327	4·4	1·1	618	2279	2009	10·9	59	9·4
CEEC mean girls<10 years	195	2·1	1·0	526	1679	1087	6·9	46	5·9
CEEC mean boys<10 years	200	2·4	1·1	554	1744	1151	7·3	49	6·4
CEEC mean girls≥10 years	280	3·6	1·0	566	2097	1640	9·7	56	8·0
CEEC mean boys≥10 years	324	4·4	1·2	631	2310	2058	11·0	62	9·5
Denmark (4–9 years, 10–17 years)	Danish National Survey of Diet and Physical Activity (DANSDA) 2011–2013								
Girls<10 years	270	5·1	2·5	906	2500	2800	8·4	210	8·9
Boys<10 years	289	5·6	2·8	1052	2700	3100	9·4	233	9·8
Girls≥10 years	254	4·3	2·4	910	2500	3000	8·3	213	9·1
Boys≥10 years	307	6·0	3·1	1183	3100	3900	10·8	249	12·4
Norway (4–9 years, 13 years)	UNGKOST 3 2015–2016								
Girls<10 years	168	4·5	3·6	729	2127	2067	6·6		
Boys<10 years	183	5·1	3·8	821	2377	2255	8·2		
Girls≥10 years	183	4·9	3·5	753	2300	2300	8·0		
Boys≥10 years	210	5·9	4·3	918	2700	2700	9·0		
North mean girls<10 years	221	4·8	3·0	820	2319	2445	7·5	210	8·9
North mean boys<10 years	237	5·3	3·3	940	2544	2691	8·8	233	9·8
North mean girls≥10 years	220	4·6	2·9	834	2403	2661	8·2	213	9·1
North mean boys≥10 years	260	6·0	3·7	1055	2906	3319	9·9	249	12·4
Austria (7–9 years; 10–14 years)	Austrian Nutrition Report (OSES) 2010–2012								
Girls<10 years	171	3·5	1·7	739	2259	3320	9·4	102	8·5
Boys<10 years	164	3·7	2·1	876	2270	3520	9·7	111	8·8
Girls≥10 years	141	3·7	1·4	683	1939	3339	8·6	88	8·1
Boys≥10 years	164	4·0	1·5	733	2214	3750	10·5	101	9·4
Belgium (3–9 years; 10–17 years)	Belgian National Food Consumption Survey (BNFCS) 2014–2015								
Girls<10 years	166	3·5	3·2	670		1645	6·8	115	
Boys<10 years	180	4·4	3·4	731		1803	7·7	118	
Girls≥10 years	183	3·6	3·3	681		1940	7·8	117	
Boys≥10 years	209	4·6	3·6	786		2406	9·7	141	
France (0–10 years; 11–17 years)	Individual National Food Consumption Survey (INCA3) 2014–2015								
Girls<10 years	228	3·5	6·4	801	2020	1691	2·4	110	6·6
Boys<10 years	243	3·7	5·5	857	2224	1860	4·6	121	7·0
Girls≥10 years	270	3·9	2·8	681	2538	2352	8·9	122	7·7
Boys≥10 years	300	5·0	3·0	786	2814	2832	10·7	146	9·6
Germany (14–18 years)	German National Nutrition Survey (Nationale Verzehrstudie) II (NVSII) 2005–2007								
Girls<10 years									
Boys<10 years									
Girls≥10 years	340	4·0	2·0	1023	3011	2471	12·1	171	9·3
Boys≥10 years	410	6·3	2·7	1277	3655	3535	15·6	231	12·7
Ireland (1–8 years; 9–17 years)	National Pre-School Nutrition Survey 2010–2011; National Children’s Nutrition Survey 2003–2004; National Teens Nutrition Survey 2005–2006								
Girls<10 years	188	4·1	2·9	789	1750	1193	7·8	156	5·6
Boys<10 years	195	4·1	3·0	808	1750	1193	8·1	156	5·8
Girls≥10 years	222	4·1	2·3	764			9·9		6·8
Boys≥10 years	322	5·6	2·7	1028			13·0		9·1
Italy (0–9·9 years; 10–17·9 years)	Third Italian National Food Consumption Survey (INRAN-SCAI) 2005–2006								
Girls<10 years		5·0	2·0	731	2235		8·6		9·0
Boys<10 years		5·0	2·0	731	2235		8·6		9·0
Girls≥10 years		4·1	2·4	770	3123		10·6		10·9
Boys≥10 years		5·6	2·6	892	2737		12·2		13·3
Netherlands (2–8 years; 9–18 years)	Dutch National Food Consumption Survey – young children (DNFCS) 2008; Dutch National Food Consumption Survey (DNFCS) 2007–2010								
Girls<10 years	117	2·7	2·2	756	2357		6·7		5·6
Boys<10 years	183	2·8	2·4	832	2362		6·9		5·9
Girls≥10 years	193	3·3	2·4	881	2562	2297	8·5	150	8·5
Boys≥10 years	233	4·1	3·0	1018	3036	2804	9·9	193	10·1
Portugal (0–9 years; 10–17 years)	National Food and Physical Activity Survey (IAN-AF) 2015–2016								
Girls<10 years	192	2·7	6·3	781	2504	1638	8·5		6·9
Boys<10 years	193	2·7	6·7	851	2539	1643	8·9		7·1
Girls≥10 years	222	4·5	3·5	757	2891	2731	16·0		9·7
Boys≥10 years	252	5·1	4·3	922	3409	3255	16·0		12·1
Spain (9–17 years)	ANIBES 2013								
Girls<10 years									
Boys<10 years									
Girls≥10 years									
Boys≥10 years									
UK (1·5–10 years; 11–18 years)	National Diet and Nutrition Survey Rolling Programme (NDNS RP) years 1–4 2008–2012								
Girls<10 years	175	3·8	1·9	780	1989	1625	7·7	134	5·9
Boys<10 years	185	4·0	2·0	807	2081	2196	8·2	141	6·2
Girls≥10 years	186	3·6	1·9	670	2065	1902	8·4	109	6·3
Boys≥10 years	233	4·4	2·4	889	2536	2960	10·7	141	8·3
West mean girls<10 years	190	3·8	3·5	768	2106	1715	6·3	122	6·9
West mean boys<10 years	206	4·0	3·3	808	2198	2026	7·2	130	7·2
West mean girls≥10 years	251	4·3	2·3	824	2640	2286	9·9	133	8·4
West mean boys≥10 years	298	5·2	2·8	1002	2948	3078	12·1	170	10·8
Europe mean girls<10 years	193	3·3	2·7	691	1974	1492	6·6	93	6·6
Europe mean boys<10 years	205	3·5	2·6	729	2062	1702	7·3	99	6·9
Europe mean girls≥10 years	259	4·1	2·0	755	2481	2095	9·8	109	8·3
Europe mean boys≥10 years	304	5·0	2·4	903	2768	2765	11·8	137	10·4

CEEC, Central and Eastern European countries.

* Where mean intakes were reported by a country for more than one age group<10 years, or more than one age group≥10 years, the number of children/adolescents surveyed in the national diet survey for each age group and sex were used to weight the reported means to produce estimate mean intakes for those aged<10 years and those aged≥10 years for each nutrient. Countries that span the 10-years boundary are: Cyprus (9–13·9 years); Ireland (9–12 years); Latvia (7–16 years); the Netherlands (9–13 years); Spain (9–12 years); Turkey (9–11 years) and the UK (4–10 years). For each nutrient regional weighted means for North, West and Central and Eastern Europe and Europe overall were calculated by weighting the<10 years and≥10 years country means shown in the table by the total child population in that country^(^^22^^–^^24^^)^.

Of the twenty-one surveys for which energy and nutrient intakes were extracted, only 38 % (*n* 8) reported intakes by socio-economic group in addition to age and sex (Estonia, France, all three Irish surveys, Dutch National Food Consumption Survey of young children, Norway, UK).

### Energy and nutrient intakes

Means reported here are estimated weighted means for Europe overall for children<10 years and≥10 years (see Tables 5 and 6 for estimated means by energy and nutrients broken down by country/survey); values in parentheses are ranges of sex and age group means provided in the survey reports. Of the nineteen macro- and micronutrients considered, no country other than Slovenia (44 %) met more than half of the WHO RNI in the nutrients and age/sex groups for which they were reported. Though patterns were evident across sex and age, there were no apparent regional trends across Europe.

#### Energy

**Fig. 1:**
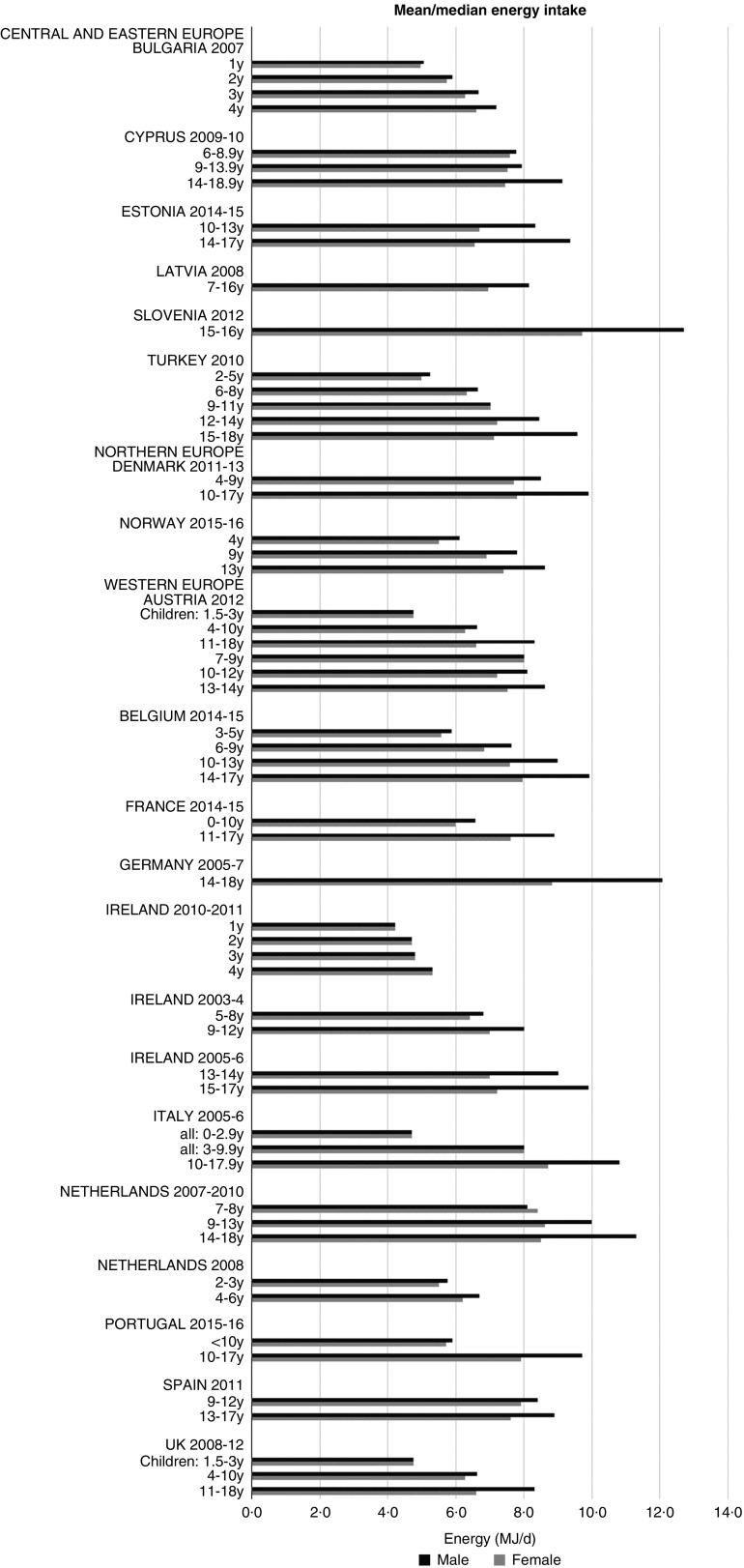
Mean/median energy intake (MJ/d) (excluding supplements). y, Years.

Although age groupings were not consistent across countries, where boys and girls were presented separately, boys’ intakes were generally higher than girls’ and older children had higher intakes (see Fig. 1). The mean energy intake was 6·0 (range 5·3–8·0) MJ and 7·7 (range 6·6–9·4) MJ for girls<10 years and≥10 years, respectively, and 6·3 (range 5·5–8·5) MJ and 9·4 (range 8·2–12·7) MJ for boys.

#### Macronutrients

**Fig. 2:**
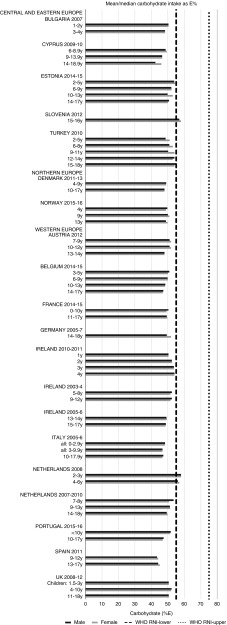
Mean/median child carbohydrate intake (percentage energy; %E) (excluding supplements). y, Years; RNI, recommended nutrient intake.

**Fig. 3:**
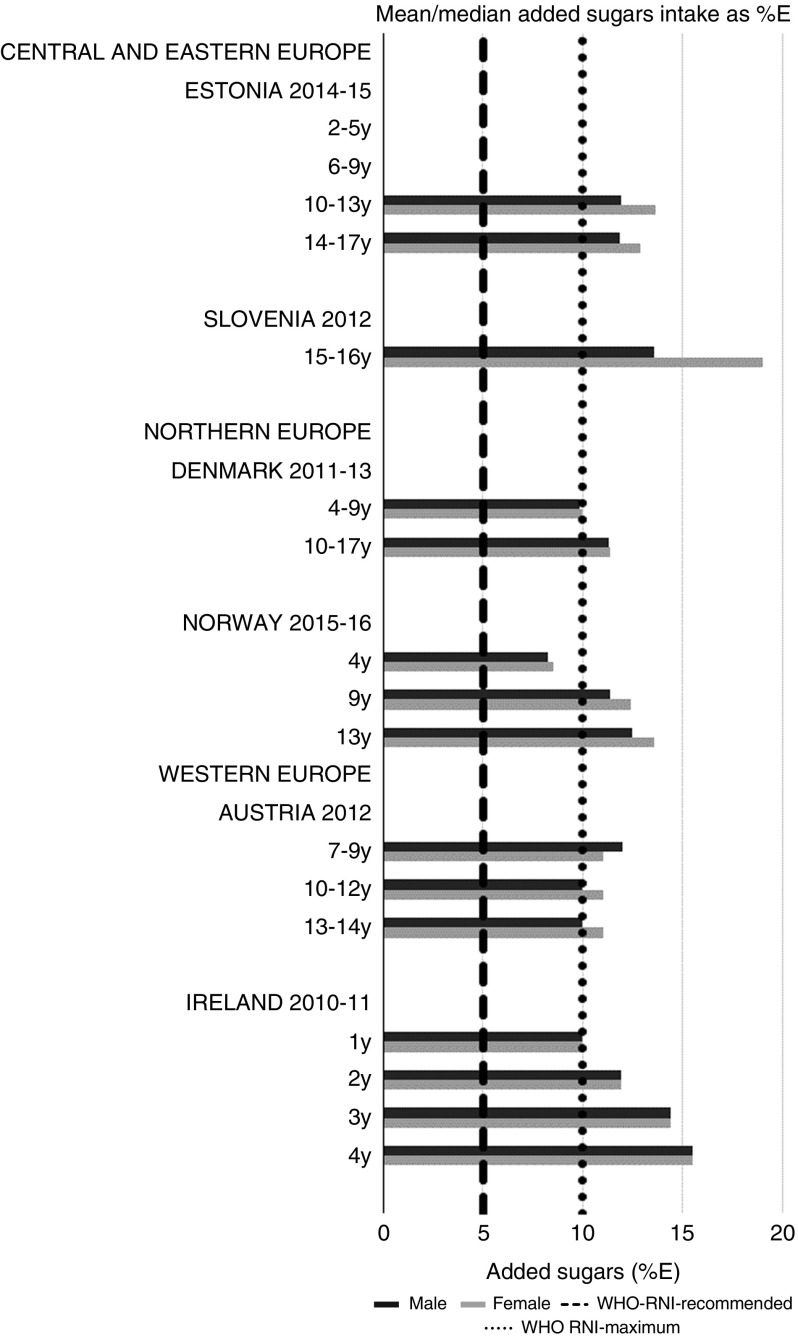
Mean/median child added sugars intake (percentage energy; %E) (excluding supplements). y, Years; RNI, recommended nutrient intake.

**Fig. 4:**
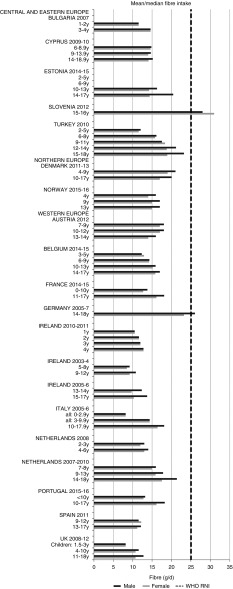
Mean/median child fibre intake (g/d) (excluding supplements). y, Years; RNI, recommended nutrient intake.

For all macronutrients, where age groups were split by sex, boys generally had higher intakes than girls in all countries except Slovenia, particularly in older children. WHO RNI^(^^15^^,^^21^^)^ attainment was universally poor in both sexes across all ages in the majority of macronutrients. The TFA RNI had the highest compliance, with all countries that reported intakes falling below the maximum value. No country fell short of the 10 %E minimum protein value and half the surveys fell between the 10 and 15 %E minimum and maximum boundaries. Only Slovenian teenagers and Dutch young children met the lower 55 %E carbohydrate RNI (Fig. 2). The mean carbohydrate intake was 183 (range 126–255) g and 233 (range 192–379) g for girls aged<10 years and≥10 years, respectively, and 192 (range 126–258) g and 281 (range 211–370) g for boys. Of the six countries that reported added sugars (*n* 6), Ireland (1 year), Denmark (4–9 years), Norway (4 years) and Austrian boys (10–12 and 13–14 years) had intakes between the recommended 5 %E and maximum 10 %E RNI and all other children exceeded the maximum (Fig. 3). Mean added sugar intakes were 46 (range 25–56) g and 58 (range 48–110) g for girls<10 years and≥10 years, respectively, and 48 (range 25–61) g and 63 (range 49–103) g for boys. Only Slovenian adolescents and German boys (14–18 years) met the 25 g fibre RNI (Fig. 4). Mean fibre intakes were 12 (range 8–19) g and 17 (range 9–31) g for girls<10 years and≥10 years, respectively, and 13 (range 8–21) g and 18 (range 11–28) g for boys.

**Fig. 5:**
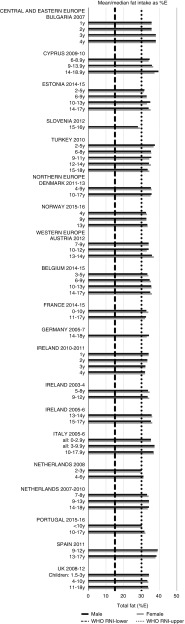
Mean/median child fat intake (percentage energy; %E) (excluding supplements). y, Years; RNI, recommended nutrient intake.

**Fig. 6:**
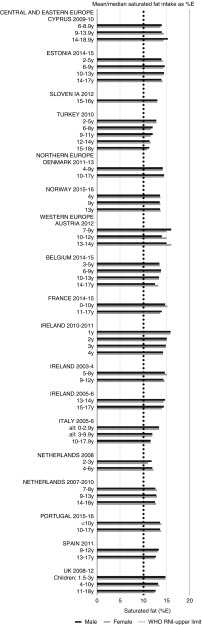
Mean/median child saturated fat intake (percentage energy; %E) (excluding supplements). y, Years; RNI, recommended nutrient intake.

**Fig. 7:**
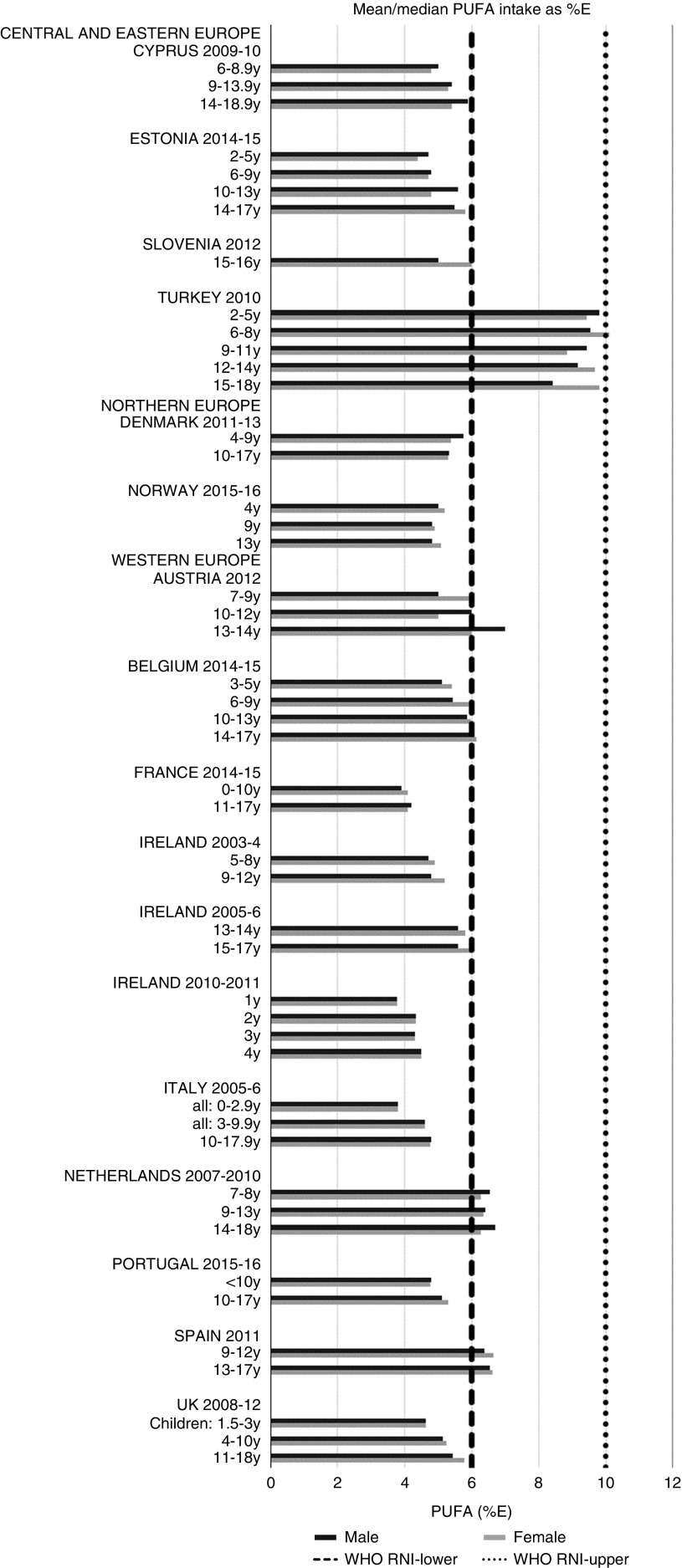
Mean/median child PUFA intake (percentage energy; %E) (excluding supplements). y, Years; RNI, recommended nutrient intake.

Total fat and saturated fats RNI compliance was particularly poor – all countries in all age groups exceeded the latter and only Slovenia and the Netherlands (2–3 years) had fat intakes below the 30 %E maximum RNI, but these were close to the upper boundary (Figs. 5 and 6). Mean fat intakes were 56 (range 38–80) g and 71 (range 60–148) g for girls<10 years and≥10 years, respectively, and 58 (range 38–80) g and 86 (range 66–177) g for boys. For saturated fats this was 21 (range 14–32) g and 25 (range 16–35) g for girls<10 years and ≥10 years, respectively, and 22 (range 14–34) g and 30 (range 18–38) g for boys. PUFA RNI attainment was mixed, although all countries except Turkey that achieved the RNI were very close to the lower 6 %E boundary (Fig. 7). Mean PUFA intakes were 10 (range 4–17) g for both sexes aged<10 years and 13 (range 9–19) g for girls and 15 (range 10–21 g) for boys aged≥10 years.

**Fig. 8:**
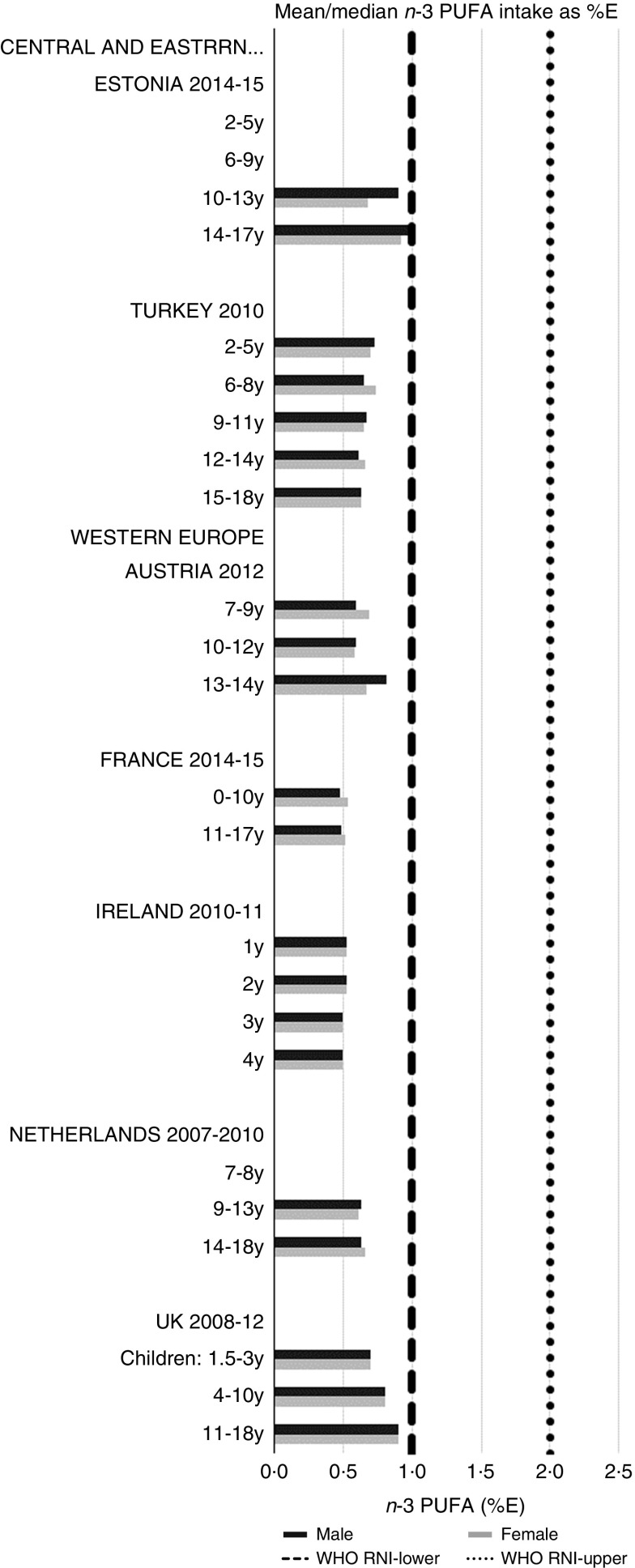
Mean/median child *n*-3 PUFA intake (percentage energy; %E) (excluding supplements). y, Years; RNI, recommended nutrient intake.

**Fig. 9:**
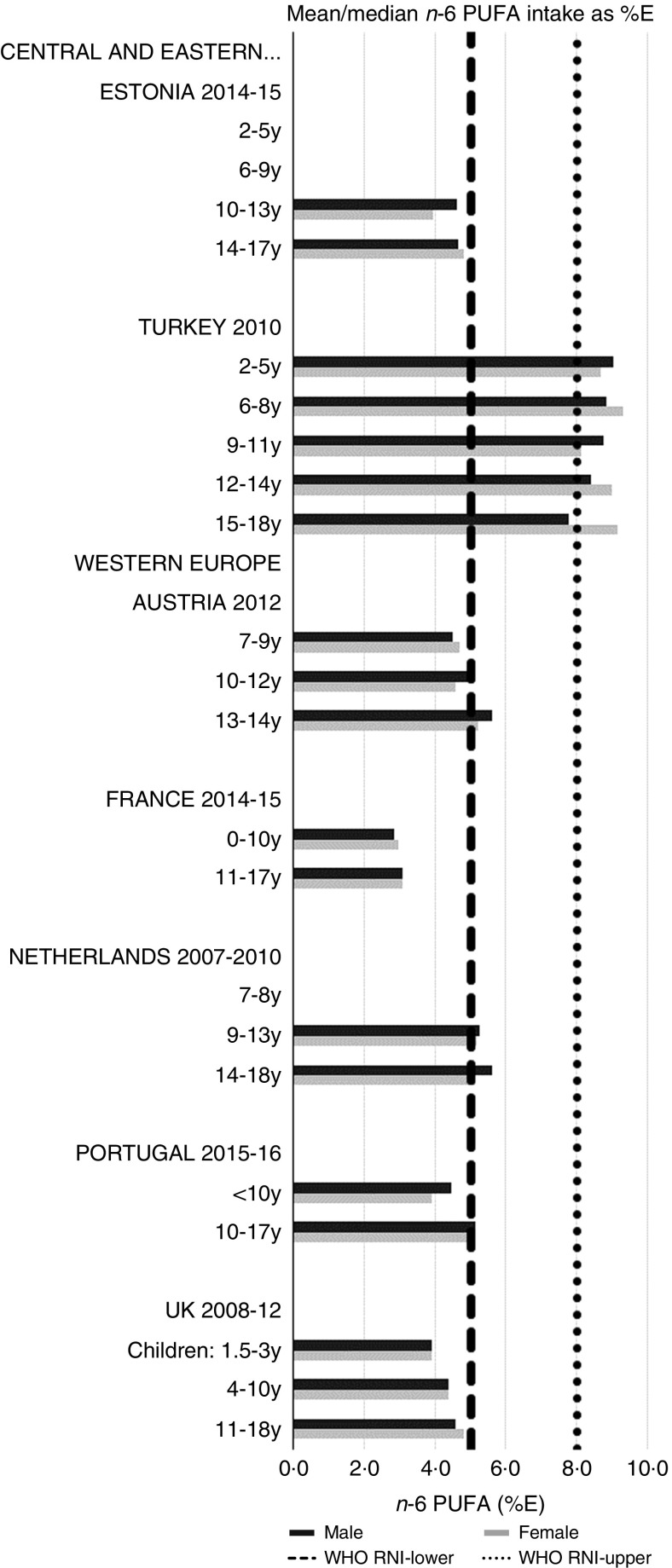
Mean/median child *n*-6 PUFA intake (percentage energy; %E) (excluding supplements). y, Years; RNI, recommended nutrient intake.

RNI attainment was relatively poor for the seven countries that reported *n*-3 and *n*-6 (omega) fat intakes; only Estonian boys (14–17 years) achieved the lower 1 %E *n*-3 RNI (Fig. 8). Just over half of countries reporting *n*-6 achieved the lower 5 %E RNI in some age categories (Fig. 9). Turkey was the only country to exceed the upper 8 %E *n*-6 limit in any age group. Mean *n*-3 intakes were 1·0 (range 0·5–1·5) g and 1·3 (range 0·5–1·6) g for girls<10 years and≥10 years, respectively, and 1·0 (range 0·5–1·4) g and 1·5 (range 0·5–2·5) g for boys. Mean *n*-6 intakes were 8·6 (range 3·0–15·6) g and 11·4 (range 3·1–17·3) g for girls<10 years and≥10 years, respectively, and 9·2 (range 2·9–15·6) g and 13·1 (range 3·1–19·7) g for boys.

#### Micronutrients

Micronutrient RNI attainment^(^^16^^–^^18^^)^ was better than for macronutrients. Micronutrient intakes are grouped according to RNI compliance and described beginning with those with greatest compliance across the countries and ending with those that demonstrate the greatest shortfall. All micronutrients except vitamin D had age-specific RNI; Fe and Zn also had sex-specific RNI for children aged 10–18 years. RNI compliance was greater in boys and younger children aged <10 years.

**Fig. 10:**
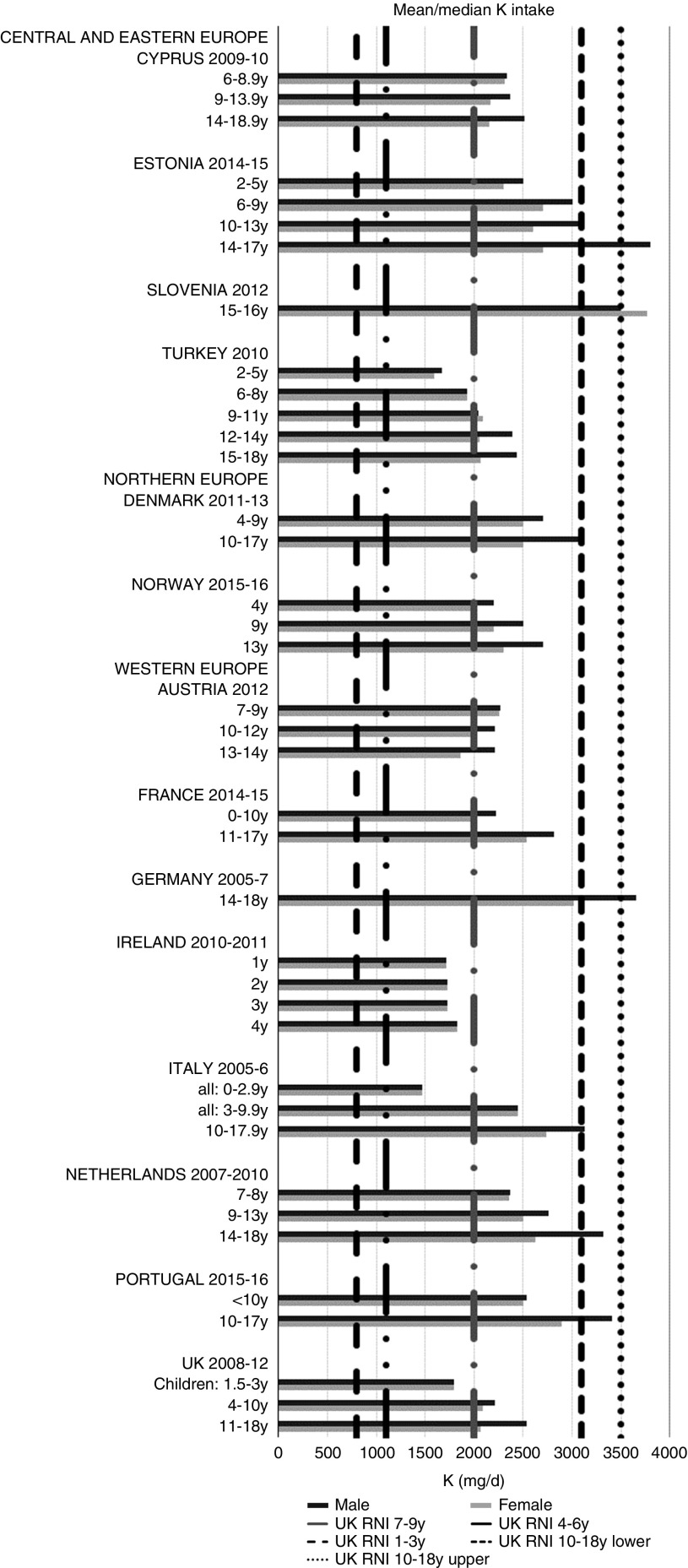
Mean/median child potassium intake (mg/d) (excluding supplements). y, Years; RNI, Reference Nutrient Intake.

**Fig. 11:**
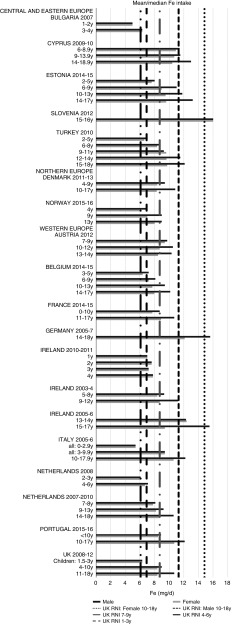
Mean/median child iron intake (mg/d) (excluding supplements). y, Years; RNI, Reference Nutrient Intake.

All countries met the vitamin B_12_ RNI across all ages, with the exception of Turkish adolescent girls. The majority of countries met the Zn RNI across the age groups surveyed; however, attainment gaps were most likely to be in adolescent girls. K and Fe intakes were mixed, but generally poorer in children aged≥10 years and girls, particularly for Fe (Figs. 10 and 11). All countries (except France) fulfilled the K RNI in some age groups and only Slovenian adolescent girls and German and Estonian adolescent boys exceeded the upper 3500 mg RNI. However, no country met the lower K RNI across all childhood stages. Mean intakes were 1974 (range 1471–2700) mg and 2481 (range 1867–3770) mg for girls<10 years and≥10 years, respectively, and 2062 (range 1471–3000) mg and 2768 (range 2039–3800) mg for boys. Bulgaria and France did not achieve the UK Fe RNI^(^^21^^)^ in any age group. In other countries lack of compliance with the Fe RNI was dominated by adolescent girls, where only Slovenia achieved the RNI. Boys had slightly higher intakes than girls – mean intakes were 6·6 (range 5·0–10·9) mg and 9·8 (range 7·7–16·0) mg for girls<10 years and≥10 years, respectively, and 7·3 (range 5·0–11·4) mg and 11·8 (range 9·0–16·0) mg for boys. However, boys have lower requirements, resulting in higher RNI attainment.

**Fig. 12:**
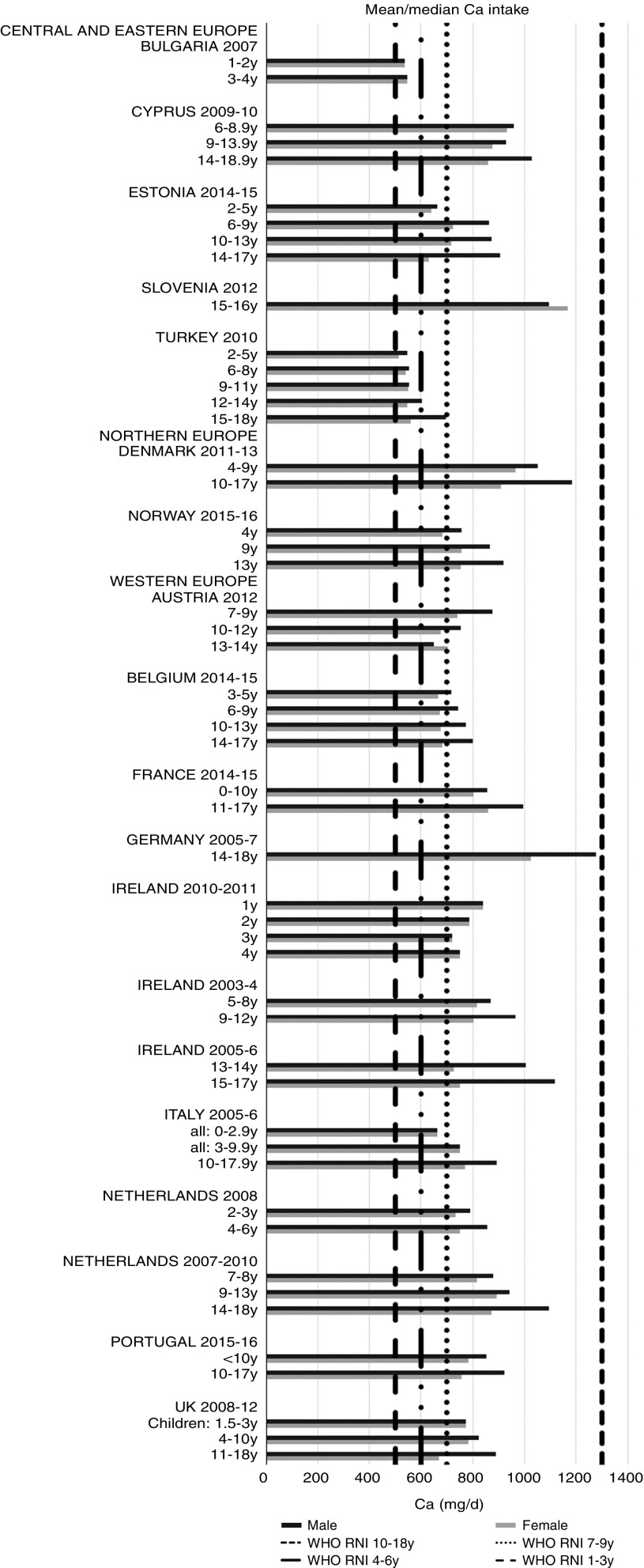
Mean/median child calcium intake (mg/d) (excluding supplements). y, Years; RNI, recommended nutrient intake.

**Fig. 13:**
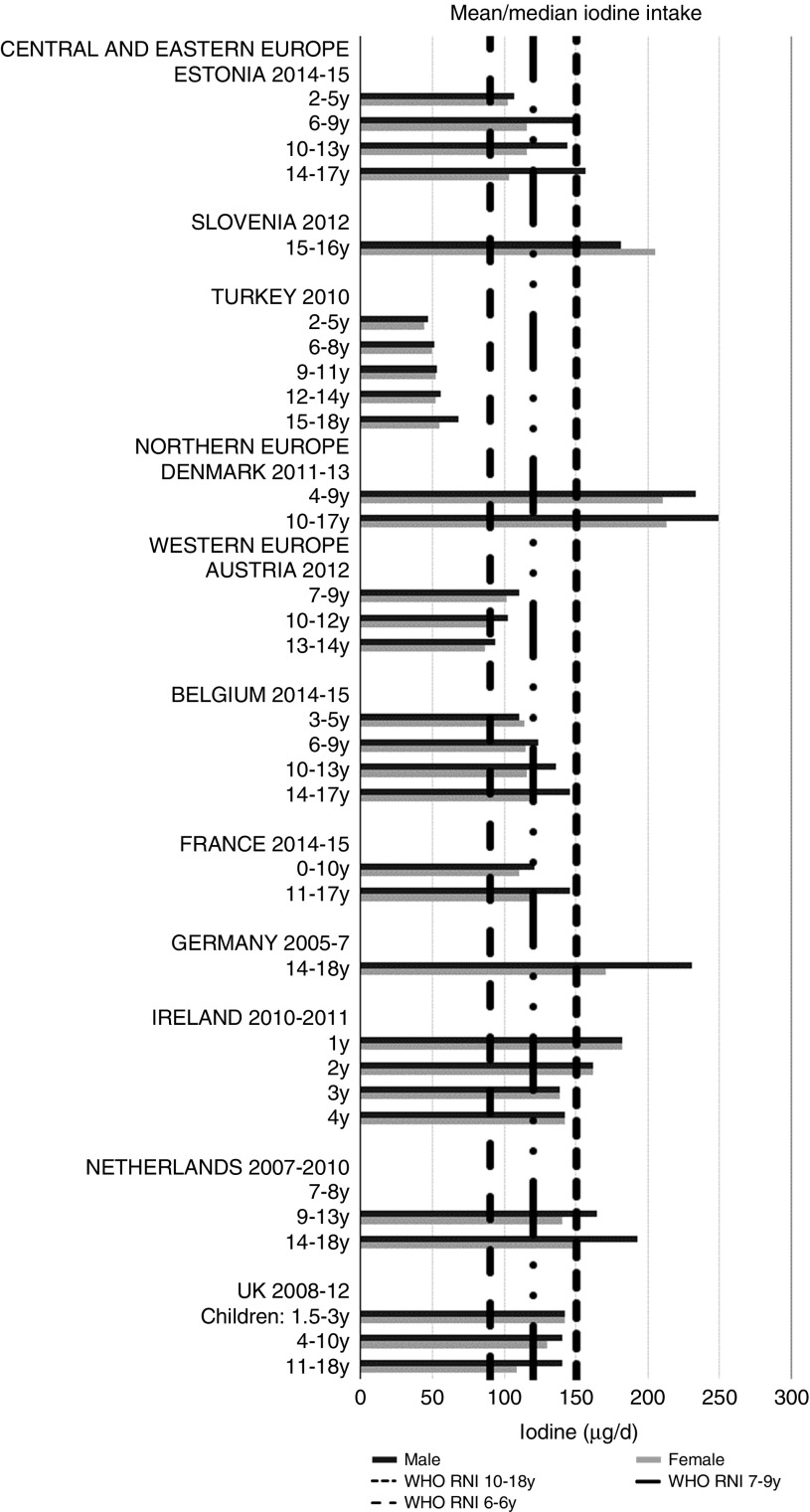
Mean/median child iodine intake (µg/d) (excluding supplements). y, Years; RNI, recommended nutrient intake.

Ca and iodine attainment was also mixed; 75 % countries reporting Ca achieved the RNI in some age groups, though no country had adequate intakes in children aged≥10 years (Fig. 12). Mean Ca intakes were 691 (range 26–1113) mg and 755 (range 545–1167 mg) for girls<10 years and≥10 years, respectively, and 729 (range 515–966 mg) and 903 (range 554–1277 mg) for boys. Three of the ten countries reporting iodine (Turkey, Austria, France) did not achieve the RNI in any age group (Fig. 13); of the remainder, attainment was spread across age groups. Mean intakes were 93 (range 44–272) µg and 109 (range 52–213) µg for girls<10 years and≥10 years, respectively, and 99 (range 47–283) µg and 137 (range 53–249) µg for boys.

**Fig. 14:**
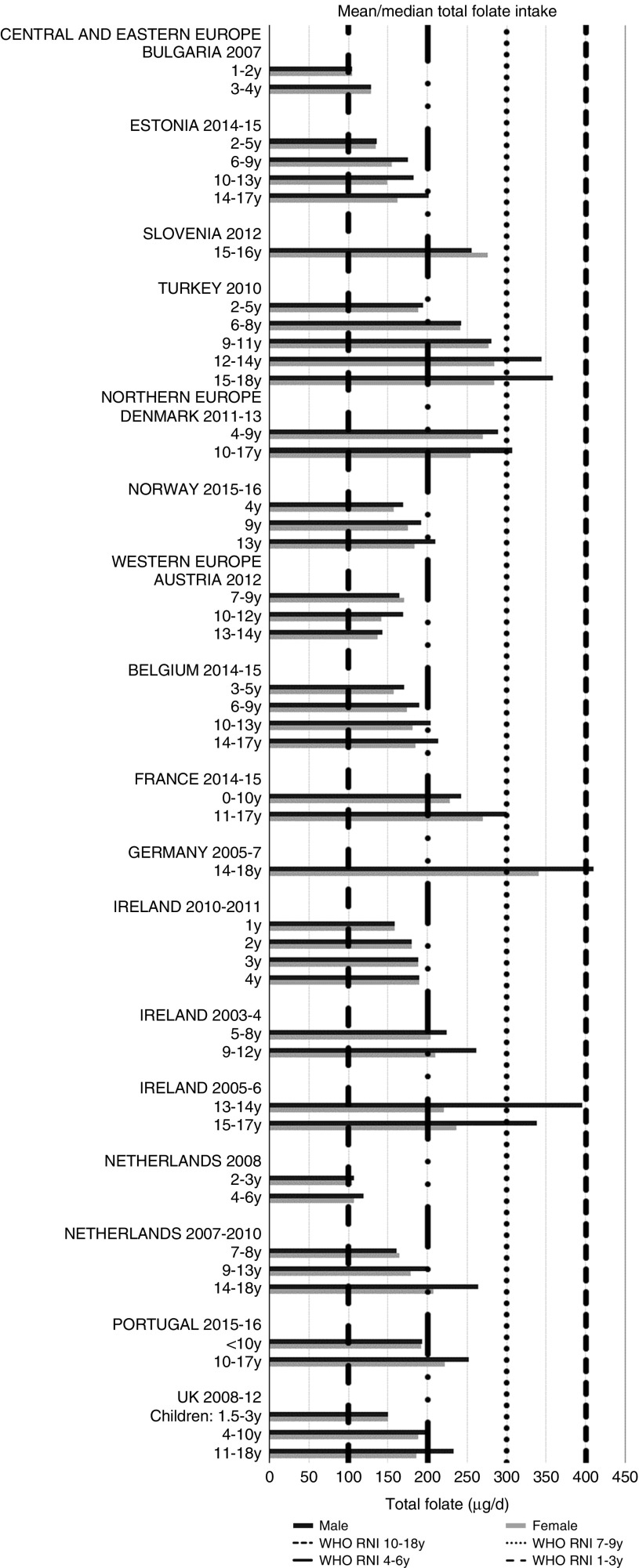
Mean/median child total folate intake (µg/d) (excluding supplements). y, Years; RNI, recommended nutrient intake.

**Fig. 15:**
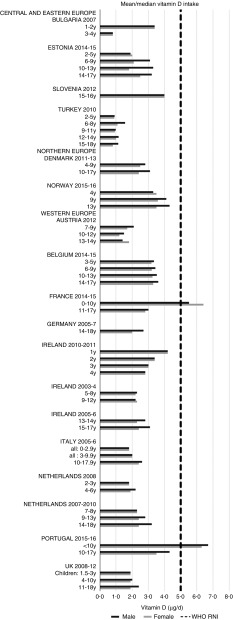
Mean/median child vitamin D intake (µg/d) (excluding supplements). y, Years; RNI, recommended nutrient intake.

**Fig. 16:**
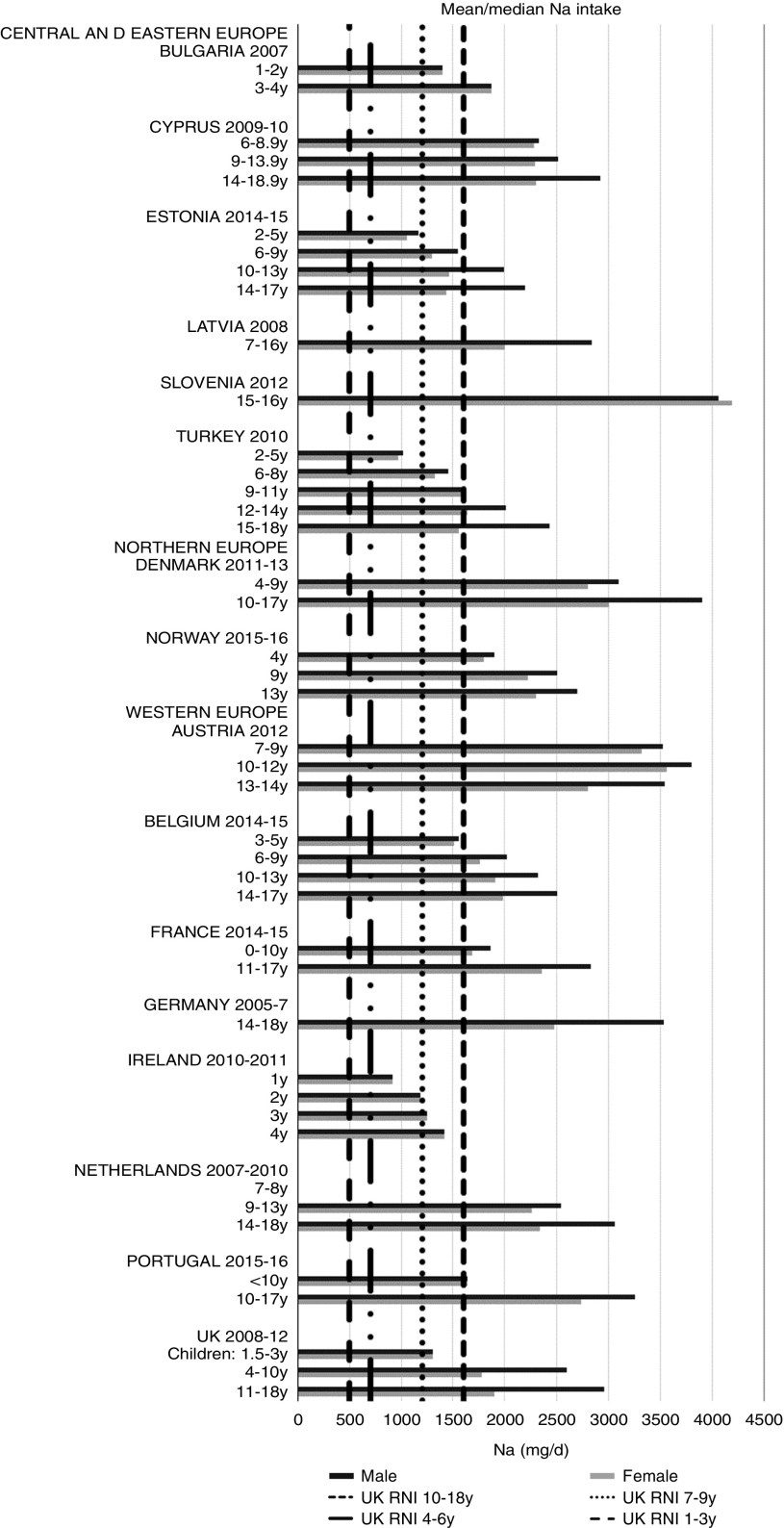
Mean/median child sodium intake (mg/d) (excluding supplements). y, Years; RNI, Reference Nutrient Intake.

Irish boys (13–14 years) were the only group aged>3 years with adequate total folate intakes (Fig. 14). Mean intakes were 193 (range 104–270) µg and 259 (range 137–340) µg for girls<10 years and≥10 years, respectively, and 205 (range 104–289) µg and 304 (range 143–410) µg for boys. The lowest RNI attainment was in vitamin D, where only French and Portuguese children aged<10 years had sufficient intakes (Fig. 15). Mean intakes were 2·7 (range 0·8–6·4) µg and 2·0 (range 0·8–4·0) µg for girls<10 years and≥10 years, respectively, and 2·6 (range 0·8–6·7) µg and 2·4 (range 1·0–4·3) µg for boys. Most countries over-consumed Na – only Estonian girls aged≥10 years and Turkish adolescent girls did not exceed the 1600 mg RNI (Fig. 16). Mean intakes were 1492 (range 918–3320 mg) and 2095 (range 1434–4191 mg) for girls<10 years and≥10 years, respectively, and 1702 (range 918–3520 mg) and 2765 (range 1599–4059 mg) for boys.

## Discussion

### Data extracted

The present review details the reporting of child intake data for energy and selected nutrients of concern in nationally representative surveys across the fifty-three countries in the WHO Europe remit^(^^1^^)^. Only a third of countries, mostly Western European, reported intake data by sex and age group. This is concerning, as potential micronutrient deficiencies may go unidentified and nutrition policies in two-thirds of the WHO Europe region, particularly outside Western Europe, may be based on limited contextual evidence that can be critical in tailoring policies to local needs. This makes impacts on other NDS and has longer-term implications for obesity; over 60 % children who are overweight before puberty are likely to remain so in adulthood^(^^25^^)^. Although Southern European countries have previously had the highest prevalence in children aged 6–9 years^(^^26^^,^^27^^)^, there has been a particularly marked increase in childhood obesity in CEEC since 2002^(^^28^^)^. In addition, six of the top ten countries for overweight and obesity in girls, and five of the top ten countries for boys aged 7–9 years in the Childhood Obesity Surveillance Initiative round 4 were CEEC^(^^29^^)^. This is concerning, as increased intakes of processed foods driven by food system changes induced by the nutrition transition in CEEC^(^^30^^)^ could begin to affect later years. If dietary data are lacking, countries may struggle to advocate and design effective policies.

Energy, and macro- and micronutrients were generally widely reported in the twenty-one surveys across eighteen countries from which intakes were extracted, though some gaps were evident. Energy was universally reported, and macronutrients slightly better represented than micronutrients. This forms a good foundation for assessing child nutrient status and identifying vulnerable age/sex groups. The largest nutrient gaps in reported intakes were TFA, *n*-3 and *n*-6 (omega) fats, added sugars and iodine, all of which have been highlighted as of concern^(^^1^^,^^31^^)^. Iodine deficiency has been linked to reduced cognitive function in children^(^^32^^)^ and remains an issue in the WHO European region. Andersson *et al.*^(^^33^^)^ examined national (about 65 %) and subnational (about 35 %) data on urinary iodine concentration and found that 43·9 % of European school-age children had insufficient intakes.

*n-*3 Fatty acids have established links with reduced blood pressure and CHD risk in adulthood, amongst other health benefits^(^^34^^,^^35^^)^, including brain development^(^^36^^)^. Over-consumption of sugar, particularly in adolescents and often from sugar-sweetened beverages, is linked to overweight and obesity via elevated energy intake and can promote suboptimal diets by displacing nutrient-rich foods^(^^28^^)^. Lack of intake data for these nutrients therefore hampers the identification of unfavourable intakes and policy formulation to prevent subsequent problems in childhood that affect the lifespan. Although there were no regional patterns in nutrient reporting, Latvia only reported on energy and Na intakes and Spain included no micronutrients. This has implications for national nutrition policies and identification of vulnerable groups in these countries.

Only a third of countries reported energy and nutrient intake by socio-economic group, by one or more indicators including education, occupation, income and social class (Table 4). This narrows opportunities to assess nutrient-based socio-economic inequalities in population subgroups, and prevents comparisons with countries that do include such stratification. Vulnerable groups may therefore be susceptible to malnutrition, with limited monitoring tools for preventative policy formation.

### Energy intakes

As expected, boys and older children had generally higher energy intakes. There were no obvious regional trends, though German and Slovenian adolescent boys had particularly high intakes, possibly due to the age range being limited to older adolescents. The literature suggests that under-reporting affects reported intakes to varying degrees across countries, making valid comparisons difficult, particularly considering that in different surveys children reported their own intake at different ages. Rothausen *et al.*^(^^37^^)^ found that misreporting in Danish children aged 7–8 years was ‘modest’, and greater in those aged 12–13 years, particularly in food diaries compared with 24 h recalls. Similarly, Lioret *et al.*^(^^38^^)^ found greater under-reporting in French children aged≥10 years than in those aged<10 years, and one study found under-reporting in British children aged 11–17 years as high as 73 %^(^^39^^)^. This suggests that the energy differential between younger and older children may be higher than that reported.

### Nutrient intakes and WHO recommended nutrient intakes status

Countries in all WHO Europe regions had poor RNI attainment levels – only Slovenia met over half of the RNI for those nutrients and age/sex groups reported (Table 4). This is concerning, as it implies that nutritional issues affect children across Europe, to an extent that may be difficult to determine due to the limitations of the data available and the gaps in data for some countries and nutrients. Older adolescents in the≥10 years range are more likely to meet RNI based on absolute levels rather than %E, such as fibre. This could explain why Slovenia had the highest percentage compliance (42 %), having generally high intakes across the nutrients extracted. This could be due to the narrow adolescent age range surveyed (15–16 years); Germany had a similar age range (14–18 years) and also had relatively high intakes. However, other countries with similar age groupings had lower intakes, supporting the possibility of the differences being genuine.

#### Macronutrients

Most countries did not meet the carbohydrate, sugar or fibre RNI in any age group. The only exceptions were German boys, who met the fibre RNI, and Slovenia, which met the total carbohydrate and fibre RNI. However, both the German and Slovenian cohorts were limited to adolescents, giving them a greater chance of having intakes high enough to meet the fibre RNI, which represents an absolute amount rather than %E and is not a child-specific target. Northern European children<10 years were more likely to meet the added sugar RNI, and other than Slovenian adolescents, Dutch children aged<10 years were the only other group to meet the lower carbohydrate RNI. This suggests that in countries where sugar data were present, most children, particularly those aged≥10 years, could be at greater risk of the weight gain and associated risks linked to high sugar and low complex carbohydrate consumption^(^^40^^)^.

Most countries had intakes indicating an unfavourable fatty acid balance across all age groups; all countries were over the upper RNI for saturated fats in all age groups and only Slovenia and very young Dutch children were below the maximum fat RNI. Dutch children aged 2–3 years were the only group with a favourable fatty acid profile, achieving the PUFA in addition to the total fat RNI. Slovenian children neither achieved the PUFA RNI nor had a substantial MUFA intake compared with other children aged≥10 years. The Netherlands and Turkey met the PUFA and *n*-6 RNI in all ages and Austria, the Netherlands and Portugal met the *n*-6 RNI in older children. Spain also met the PUFA RNI in all ages and Cyprus, Italy and Spain had relatively high MUFA intakes. The favourable intakes in these countries could indicate aspects of a Mediterranean diet pattern, which when adhered to in its complete form and supported by other factors such as physical activity, has been linked to reduced childhood obesity^(^^41^^)^. Conversely, *n*-3 intakes were poor, with only Estonian adolescent boys achieving the RNI.

TFA had the highest RNI compliance for those countries which reported it. This may reflect positive moves to reduce levels in the food supply following advice from health bodies like the WHO^(^^1^^)^, including bans, labelling legislation and voluntary product reformulation^(^^42^^–^^44^^)^. However, the low number of countries reporting TFA demonstrates the need for a common and comprehensive blueprint for conducting NDS and gathering nutrient intake data across Europe.

#### Micronutrients

As with macronutrients, there were no clear regional patterns in micronutrient intakes or RNI attainment. However, compliance was highest in boys and children aged<10 years. Unlike macronutrients, micronutrient RNI are based on absolute intakes rather than %E. Yet although most micronutrients have different RNI for specific age groups, intakes in children aged≥10 years were generally not sufficient to meet RNI for older children, particularly girls. Even in Zn and vitamin B_12_, where RNI attainment was high, shortfalls in adolescent girls were apparent, highlighting them as a vulnerable group.

Although not the worst in overall RNI attainment, Fe was a particular issue for adolescent girls, with all countries except Slovenia having inadequate intakes. This is consistent with previous (non-national) European-based reviews and relates to higher requirements, primarily due to menstruation^(^^45^^,^^46^^)^. Adolescent girls are at greater risk of Fe-deficiency anaemia, and deficiency is associated with reduced intellectual, immune and other metabolic functions^(^^46^^)^. Deficiency in this group may also be underestimated, as UK RNI were used instead of WHO RNI because the latter have different values for different bioavailabilities and menarchal status, which would be difficult to determine^(^^16^^)^. However, although agreement between the UK and WHO RNI was good for children aged<10 years, WHO RNI requirements are much higher for girls post-menarche, even when using the RNI that assumes the highest bioavailability from diet (15 %)^(^^16^^,^^21^^)^. The scale of European deficiency in this group may therefore be greater than previously thought, and policy initiatives may be required to improve Fe intakes.

Ca intakes were inadequate in older boys and girls. Ca is needed for bone and tooth development, metabolic processes including muscle and nerve function and its metabolism is linked with vitamin D intake. Vitamin D intakes, assessed by a singular absolute amount, rather than age-specific RNI, were universally lacking other than in younger Portuguese children. This is an important issue, as in addition to roles in bone, muscle and immune function, deficiency is linked to rickets^(^^47^^,^^48^^)^. Although rickets was of relatively little concern in Europe in the latter half of the 20th century, in recent years prevalence has risen, particularly in the UK and Northern European countries and for individuals with darker skin or who cover up for religious and other reasons, as less can be synthesised on exposure to sunlight^(^^49^^)^.

Total folate intakes were universally poor, with no children aged>3 years achieving the RNI. Na intakes exceeded the RNI in all children except adolescent girls from Estonia and Turkey, which are both CEEC. This is despite the potential for under-reporting due to intakes being derived from self-assessed dietary methodologies rather than 24 h urinary biomarkers. Further efforts are needed to promote the consumption of low-salt, minimally processed foods and advance reformulation of foods commonly consumed by children – these will vary by country, but might include bread, cheeses and breakfast cereals. However, salt iodisation is a primary means of increasing population iodine intakes, and iodine was the least reported micronutrient. Calls to reduce salt intake can lead to questions of compatibility with iodine intake goals, especially in CEEC. With almost half of European school-age children having insufficient intakes^(^^33^^)^, which can cause reduced cognitive function^(^^32^^)^, care is needed in approaches to tackle Na over-consumption, especially where iodine RNI attainment is low and salt iodisation is practised^(^^50^^)^. However, evidence is clear that appropriate Na and iodine intakes can be achieved in the context of Na reduction initiatives^(^^18^^)^, as iodine concentration in salt can be increased or alternative vehicles for iodine sourced.

Of the micronutrients investigated, our findings show that Fe, vitamin D, total folate and Na would benefit from European-wide policy focus to improve intakes, particularly in girls and children aged≥10 years. Effective food-based approaches, including product reformulation and fortification, currently exist alongside targeted supplementation for at-risk groups. Aside from total folate, the WHO Europe Food and Nutrition Action Plan^(^^1^^)^ identifies these as nutrients of concern, although this refers to all ages rather than specifically children. The Action Plan also highlights energy, saturated fat and sugar reduction as priorities and recommends a suite of policy options to address their excess intake, which our findings support. However, the plan does not discuss the increase of carbohydrate, fibre or *n*-3 and *n*-6 (omega) fats, and our findings show that the RNI for these were often not met. Countries across WHO Europe should also be encouraged to address this in policy and guidance, for example increased use of whole grains in manufactured products or public education on sources of *n*-3 and *n*-6 (omega) fats.

### Strengths and limitations

This review presents a much-needed up-to-date review of national child energy and nutrient intakes across Europe. It also reports intakes against WHO RNI, enabling governments and policymakers to better use NDS to inform initiatives to improve diets and reduce diet-related diseases in groups and areas of greatest need. It is well documented that energy, macronutrient and Na over-consumption is linked to childhood obesity and related NCD^(^^1^^)^ and poor micronutrient intakes continue to cause health problems in children^(^^45^^,^^46^^,^^49^^)^. Blundell *et al.*^(^^51^^)^ found>10 % inter-country variation in obesity prevalence and cited differences in national age profiles and sociodemographic patterns as key contributors. The present review highlights both the scale and the potential hidden extent of such issues, showing that reported lack of compliance with WHO RNI may be the tip of the iceberg, with many countries’ intakes unknown. In addition to previous reviews, which document NDS provision across Europe^(^^6^^,^^12^^)^, the present review also highlights whether and how surveys report nutrient intakes by socio-economic group, helping to direct further research in this area.

A primary limitation is that inter-country comparisons are difficult, as age groupings were inconsistent. The most extreme example of this drawback was in Andorra, which could not be included in the present review as the lowest age group included both adults and children. Age groupings also differed within countries; Bulgaria split children into four groups for energy, but two for other macro- and micronutrients, making consistent and complete analysis difficult. In addition, several countries did not separate girls and boys in the youngest age groups. Raw survey data could be used in future work to create consistent age groups and obtain more reliable conclusions.

Differences in the reporting of nutrient intakes across and within countries further hindered comparisons and, in some cases, limited RNI assessment. For example, Estonia did not report nutrients in all age groups and the three Irish surveys reported different nutrients. Bulgaria reported some nutrients by %E and others with absolute values; because age groups for energy differed from other nutrients, the %E needed to assess macronutrient RNI could not always be calculated, resulting in knowledge gaps. Age groups did not always correspond with RNI age boundaries, particularly in micronutrients, making it difficult to assess attainment. However, examples in the literature exist where international comparisons are made despite different age groupings^(^^7^^)^. Using RNI to assess nutrient intake adequacy also has limitations, as assertions are only as good as the data on which they are based, which may be incomplete. RNI are estimates of the amount of a nutrient needed to ensure that the needs of the majority of a group (97·5 %) are being met; therefore RNI err on the side of caution and may over-estimate inadequacies. The proportion of intakes in a population group below the estimated average requirements is a more appropriate measure of nutrient inadequacy than the proportion below the RNI; however, lack of raw data from sufficient countries prevented this^(^^16^^)^. Additionally, although the<10 years and≥10 years age group splits were chosen to align with the RNI age cut-offs, different cut-offs will have produced different results. Despite these difficulties, the present review remains an important study that displays nutrient intakes in children, which the WHO defines as a vulnerable group^(^^1^^)^. Any difficulties posed by lack of comparability serve to highlight the pressing need for harmonisation of methodologies and approaches.

The country means (Tables 5 and 6) for the<10 years or≥10 years groups are approximations that depend on the age ranges surveyed. For instance, the Slovenian NDS covered a small age range (15–16 years); reported mean intakes may therefore be less representative of the≥10 years group than countries that have surveyed a wider age range. Similarly, the contribution to the weighted estimated means for its region and Europe overall may be unrepresentative. The country-specific means for countries with multiple age groups above or below the 10-year cut-off are approximations based on the assumption that the numbers surveyed in each age group are proportionate to those in the total child population, the latter being used due to availability. Additionally, in some countries age ranges spanned the 10-year boundary, though broadly speaking the majority of children could be allocated to either the<10 years or≥10 years group.

The different dietary assessment methodologies used by the surveys also limit the validity of comparisons. As under-reporting is common and varies across methods and is affected by multiple other factors, the impact on reported intakes differs across countries and compounds difficulties in making comparisons. This is exacerbated by the exclusion of under-reporters by some countries (Austria, France, Norway), whereas other countries include under-reporters (Cyprus, Denmark, Ireland, Italy, the Netherlands, Slovenia and the UK) and the remainder did not specify.

Discrepancies in national food composition databases create further compatibility issues. The present review used sucrose as a proxy for added sugars, as surveys typically did not distinguish between the two. Consequently, intakes may differ as the number of mono- and disaccharides included varies. Not all surveys had available user guides to determine the methods used to derive nutrient values. With fibre, the Englyst method usually generates lower results than the AOAC for certain cereals, fruits, white beans and groundnuts^(^^52^^)^. Certain micronutrients may also be derived from a narrow range of foods, making them less valid in representing population intakes. Similarly, databases do not address fortification in a common manner, as with iodine^(^^53^^)^. This is problematic, as the severity of identified deficiencies may be misrepresented.

Future work could explore raw survey data to create common age groups and minimise the impact of inconsistencies. This would help determine whether extremes such as Slovenian macronutrient intakes are genuine differences or due to the age range covered. It would also allow the alignment of age groups with WHO RNI, increasing the accuracy of identifying deficiencies.

## Conclusion

This review reported macro- and selected micronutrient intakes in children across WHO Europe using the latest available reported NDS intakes and assessed these against WHO RNI. Energy and nutrient intakes were extracted from twenty-one surveys covering a third (eighteen), mainly Western, WHO European countries. Most countries reported intakes from a good range of nutrients, particularly macronutrients, so where nutrient intakes were reported, countries generally had a sound basis to assess child nutrient status. However, TFA, *n*-3 and *n*-6 (omega) fats, added sugar and iodine were least reported. These gaps are concerning, as potential deficiencies could go undetected and nutrition policies implemented could be based on limited evidence. WHO RNI attainment was generally poor – most countries met under half of the RNI for the nutrients and age/sex groups reported, implying that widespread nutrition issues could exist across Europe. Macronutrient RNI compliance was universally poor, and although micronutrients were slightly better, attainment was worse in girls and children≥10 years. Fat and saturated fats, vitamin D, Na, total folate and Fe had the lowest compliance. Only eight countries reported intakes by socio-economic group and different indicators were used. This narrows opportunities to assess inequalities and vulnerable groups susceptible to malnutrition and limits the monitoring tools available for policy formation. Different age groups, methodologies, nutrient composition databases and under-reporting are the main limitations, potentially misrepresenting true intakes and preventing inter-country comparisons. Future work could use raw NDS data to conduct stratified analyses with consistent age groups. Governments and health bodies should continue efforts to encourage European countries to report a full range of nutrient intakes by various sociodemographic variables in a standardised format.

## Appendix 1. Reported mean macronutrient intakes for children and adolescents in European national dietary surveys

**Table A1:** ■

Country	Survey	Year	Energy (MJ)	Energy (kcal)	Protein (g)	CHO (g)	Sugars (g)	Sucrose (g)	Fibre (g)	Total fat (g)	Saturated fats (g)	MUFA (g)	PUFA (g)	TFA (g)	*n*-3 (g)	*n*-6 (g)
Austria	Austrian Nutrition Report (OSES)	2010–2012														
	Female: 7–9 years		8·0	1910	62	248		53	17·0	72	31·8	23·3	12·7		1·5	10·0
	Female: 10–12 years		7·2	1731	61	225		48	17·0	63	28·9	21·2	9·6		1·1	8·8
	Female: 13–14 years		7·5	1783	67	214		49	14·0	73	31·7	23·8	11·9		1·3	10·3
	Male: 7–9 years		8·0	1920	62	245		58	18·0	73	34·1	23·5	10·7		1·3	9·6
	Male: 10–12 years		8·1	1940	68	247		49	18·0	73	30·2	23·7	12·9		1·3	10·8
	Male: 13–14 years		8·6	2058	72	247		51	16·0	82	34·3	27·4	16·0		1·9	12·8
Belgium	Belgian Food Consumption Survey 2014–2015	2014–2015														
	Female: 3–5 years		5·6	1329	46	166	92		12·9	50	20·0	18·0	8·0	0·6		
	Female: 6–9 years		6·8	1633	56	204	105		14·2	64	25·0	23·0	11·0	0·7		
	Female: 10–13 years		7·6	1812	63	219	107		15·2	72	27·0	26·0	12·0	0·7		
	Female: 14–17 years		8·0	1904	66	223	106		15·8	76	28·0	28·0	13·0	0·8		
	Male: 3–5 years		5·9	1406	48	179	100		12·3	52	21·0	18·0	8·0	0·6		
	Male: 6–9 years		7·6	1824	63	227	120		14·3	70	28·0	26·0	11·0	0·8		
	Male: 10–13 years		9·0	2149	75	260	131		15·9	85	32·0	31·0	14·0	0·9		
	Male: 14–17 years		9·9	2369	83	280	135		17·0	92	33·0	34·0	16·0	0·9		
Bulgaria	National Survey on Nutrition of Infants and Children Under 5 and Family Child Rearing 2007	2007														
	Female: 1 year		5·0	1185	43	159	27		11·5	50	14	10·4	8·4			
	Female: 2 years		5·7	1370	43	159	27		11·5	50	14	10·4	8·4			
	Female: 3 years		6·3	1496	51	191	35		14·6	68	15	11·7	10·3			
	Female: 4 years		6·6	1579	51	191	35		14·6	68	15	11·7	10·3			
	Male: 1 year		5·0	1206	43	159	27		11·5	50	14	10·4	8·4			
	Male: 2 years		5·9	1409	43	159	27		11·5	50	14	10·4	8·4			
	Male: 3 years		6·7	1592	55	191	35		14·6	68	15	11·7	10·3			
	Male: 4 years		7·2	1718	55	191	35		14·6	68	15	11·7	10·3			
Cyprus	A study of the dietary intake of Cypriot children and adolescents aged 6–18 years	2009–2010														
	Female: 6–8·9 years		7·6	1811	73	223			14·6	69	27·6	30·4	9·7			
	Female: 9–13·9 years		7·5	1793	74	209			14·0	73	28·7	32·9	10·6			
	Female: 14–18·9 years		7·5	1781	73	205			14·1	74	28·3	33·6	10·7			
	Male: 6–8·9 years		7·8	1856	75	226			14·8	72	28·9	31·3	10·3			
	Male: 9–13·9 years		7·9	1898	82	221			14·7	76	29·5	34·0	11·4			
	Male: 14–18·9 years		9·1	2180	96	231			15·2	96	37·1	43·8	14·3			
Denmark	Danish Dietary Habits 2011–2013	2011–2013														
	Female: 4–9 years		7·7	1840	64	220		46	19·0	73	29·0	26·0	11·0	1·2		
	Female: 10–17 years		7·8	1864	67	222		53	17·0	73	30·0	26·0	11·0	1·1		
	Male: 4–9 years		8·5	2032	71	243		50	21·0	80	32·0	28·0	13·0	1·3		
	Male: 10–17 years		9·9	2366	90	277		67	20·0	94	38·0	34·0	14·0	1·4		
Estonia	National Dietary Survey	2014–2015														
	Female: 2–5 years				48											
	Female: 6–9 years				56			56			26·7	20·9	8·7	0·5	1·3	6·7
	Female: 10–13 years		6·7	1602	54	214		55	14·2	61	26·6	20·4	8·9	0·5	1·2	7
	Female: 14–17 years		6·6	1568	56	199		51	14·4	63	25·3	22·3	10·6	0·4	1·6	8·4
	Male: 2–5 years				51											
	Male: 6–9 years				67			61			30·4	23·7	9·9	0·6	1·4	7·6
	Male: 10–13 years		8·3	1993	73	252		59	16·3	79	32·8	27·9	12·9	0·6	2	10·2
	Male: 14–17 years		9·4	2242	85	288		66	20·4	87	35·7	30·8	14·7	0·6	2·5	11·6
France	INCA3	2014–2015														
	Female: 0–10 years		6·0	1433	53	176	95		12·7	54	24·3	17·8	6·4		0·9	4·7
	Female: 11–17 years		7·6	1818	70	226	98		16·1	66	27·9	22·4	8·5		1·0	6·2
	Male: 0–10 years		6·6	1574	58	199	103		13·8	57	25·7	18·9	6·6		0·8	5·0
	Male: 11–17 years		8·9	2123	83	262	111		18·1	77	33·0	26·1	9·9		1·1	7·3
Germany	German National Nutrition Survey II	2005–2007														
	Female: 14–18 years		8·8	2108	66	274			23·2	77						
	Male: 14–18 years		12·1	2883	94	355			26·0	110						
Ireland	National Pre-school Nutrition Survey	2010–2011														
	1 year		4·2	1005	39	126	70	25	10·5	38	17·7	13·6	4·2		0·6	
	2 years		4·7	1122	43	146	74	33	11·6	42	18·8	14·0	5·4		0·7	
	3 years		4·8	1148	43	154	76	41	12·0	41	18·9	13·8	5·5		0·6	
	4 years		5·3	1264	47	171	84	49	12·8	45	20·0	15·2	6·3		0·7	
Ireland	National Children’s Food Survey	2003–2004														
	Female: 5–12 years		6·7	1601	54	217			8·8	61	26·2	20·8	9·0			
	Female: 5–8 years		6·4	1517	52	208			8·5	58	25·6	19·6	8·3			
	Female: 9–12 years		7·0	1654	56	227			9·2	63	26·9	21·9	9·6			
	Male: 5–12 years		7·4	1759	60	246			10·0	66	28·4	22·5	9·4			
	Male: 5–8 years		6·8	1625	55	226			9·2	61	26·5	20·6	8·6			
	Male: 9–12 years		8·0	1890	64	264			10·8	70	30·3	24·3	10·2			
Ireland	National Teens’ Food Survey	2005–2006														
	Female: 13–17 years		7·1	1696	60	222			10·1	68	27·2	24·4	11·1			
	Female: 13–14 years		7·0	1674	59	220			9·7	67	27·0	24·2	10·7			
	Female: 15–17 years		7·2	1712	61	223			10·3	69	27·3	24·5	11·5			
	Male: 13–17 years		9·5	2256	86	293			13·1	89	36·7	31·6	14·0			
	Male: 13–14 years		9·0	2137	82	277			12·3	85	35·3	29·7	13·2			
	Male: 15–17 years		9·9	2344	88	304			13·7	92	37·7	33·0	14·7			
Italy	Third Italian National Food Consumption Survey INRAN-SCAI	2005–2006														
	All: 0–2·9		4·7	1113	42	147	71		8·2	44	16·6	19·1	4·7			
	All: 3–9·9		8··0	1914	74	240	86		14·4	80	25·4	37·0	9·8			
	Female: 10–17·9		8·7	2091	82	263	88		16·4	86	26·8	40·3	11·1			
	Male: 10–17·9		10·8	2576	99	327	108		18·1	105	33·1	49·0	13·7			
Latvia	Latvian National Food Consumption Survey 2007–2009	2007–2009														
	Female: 7–16 years		6·9	1660												
	Male: 7–16 years		8·2	1948												
Netherlands	Dutch National Food Consumption Survey – young children (DNFCS) 2008	2008														
	Female: 2–3 years		5·5	1308	43	187	119		12·0	43	16·0			1·1		
	Female: 4–6 years		6·2	1479	46	209	129		13·0	51	20·0			1·4		
	Male: 2–3 years		5·8	1375	44	196	124		13·0	46	18·0			1·2		
	Male: 4–6 years		6·7	1587	51	222	135		14·0	55	21·0			1·4		
Netherlands	Dutch National Food Consumption Survey (DNFCS) 2007–2010	2007–2010														
	Female: 7–8 years		8·4	2011	51	255	140		15·0	76	29·0	17·0	8·0			
	Female: 9–13 years		8·6	2042	63	262	141		15·9	78	29·4	20·0	9·0		1·4	11·7
	Female: 14–18 years		8·5	2028	68	253	127		17·5	75	28·7	23·0	10·0		1·5	11·6
	Male: 7–8 years		8·1	1929	56	258	141		16·0	71	27·0	18·0	8·0			
	Male: 9–13 years		10·0	2275	74	292	154		17·8	86	32·3	23·0	10·0		1·6	13·3
	Male: 14–18 years		11·3	2690	83	332	164		21·4	102	37·3	27·0	11·0		1·9	16·7
Norway	UNGKOST 3	2015–2016														
	Female: 4 years		5·5	1315	51	158		28	14·0	50	21·0	17·0	8·0			
	Female: 9 years		6·9	1649	63	207		51	15·0	60	25·0	20·0	9·0			
	Female: 13 years		7·4	1769	68	219		60	15·0	66	27·0	23·0	10·0			
	Male: 4 years		6·1	1458	56	176		30	16·0	54	23·0	18·0	8·0			
	Male: 9 years		7·8	1864	74	228		53	17·0	68	28·0	23·0	10·0			
	Male: 13 years		8·6	2055	83	247		64	17·0	76	31·0	27·0	11·0			
Portugal	National Food and Physical Activity Survey (IAN-AF)	2015–2016														
	Female: <10 years		5·7	1361	57·9	175	89		12·8	46	21·0	20·5	7·2	0·7		5·9
	Female: 10–17 years		7·9	1872	82·8	219	88		16·1	67	28·7	27·0	11·0	1·2		10·5
	Male: <10 years		5·9	1392	56·2	180	90		13·2	47	21·0	20·9	7·4	0·7		6·9
	Male: 10–17 years		9·7	2303	103·5	273	107		18·2	81	34·9	31·8	13·1	1·5		13·1
Slovenia	Dietary Intake of Macro- and Micronutrients in Slovenian Adolescents	2012														
	Female: 15–16 years		9·7	2312	86	379	195	110	31·0	82	35·0	29·0	17·0			
	Male: 15–16 years		12·7	3043	96	370	170	103	28·0	82	36·0	30·0	16·0			
Spain	ANIBES	2013														
	Female: 9–12 years		7·9	1893	73	209	88		12·2	82	27·5	33·6	14·0			
	Female: 13–17 years		7·6	1823	71	206	87		11·2	77	25·2	31·2	13·4			
	Male: 9–12 years		8·4	2006	81	218	94		11·5	87	29·6	35·8	14·2			
	Male: 13–17 years		8·9	2124	85	235	91		12·1	91	30·0	37·3	15·4			
Turkey	Turkey Nutrition and Health Survey 2010 (TNHS)	2010														
	Female: 2–5 years		5·0	1190	37	148			11·5	49	16·9	15·8	12·5		0·9	11·5
	Female: 6–8 years		6·3	1510	45	193			15·8	60	19·6	19·0	16·9		1·2	15·6
	Female: 9–11 years		7·0	1679	51	218			18·3	64	21·5	21·8	16·5		1·2	15·1
	Female: 12–14 years		7·2	1723	51	221			18·8	68	22·1	22·6	18·5		1·3	17·2
	Female: 15–18 years		7·1	1701	49	221			18·9	65	20·5	21·6	18·6		1·2	17·3
	Male: 2–5 years		5·2	1253	39	152			12·0	52	17·9	16·9	13·6		1·0	12·6
	Male: 6–8 years		6·6	1587	49	202			16·1	62	21·1	20·1	16·8		1·1	15·6
	Male: 9–11 years		7·0	1677	52	211			17·5	66	22·4	21·8	17·6		1·2	16·3
	Male: 12–14 years		8·4	2017	62	261			21·1	77	25·5	25·4	20·5		1·4	18·9
	Male: 15–18 years		9·6	2288	68	300			23·2	85	28·7	29·2	21·4		1·6	19·7
UK	National Diet and Nutrition Survey (NDNS) Years 1–4	2008–2012														
	Children: 1·5–3 years		4·8	1126	43	151	76		8·2	43	18·5	14·4	5·8	0·8	0·9	4·9
	Female: 4–10 years		6·3	1489	53	205	95		10·7	56	22·3	20·0	8·7	1·1	1·3	7·4
	Female: 11–18 years		6·6	1569	56	211	90		10·7	60	21·7	22·7	10·1	1·1	1·6	8·5
	Male: 4–10 years		6·6	1573	57	219	100		11·5	58	23·0	21·0	9·0	1·1	1·4	7·6
	Male: 11–18 years		8·3	1972	74	265	116		12·8	74	27·8	27·6	11·9	1·4	1·9	10·0

CHO, carbohydrates; TFA, *trans*-fatty acids.

## Appendix 2. Reported mean micronutrient intakes for children and adolescents in European national dietary surveys

**Table A2:** ■

Country	Survey	Year	Total folate (µg)	Vitamin B_12_ (µg)	Vitamin D (µg)	Ca (mg)	K (mg)	Na (mg)	Fe (mg)	Iodine (µg)	Zn (mg)
Austria	Austrian Nutrition Report (OSES)	2010–2012									
	Female: 7–9 years		171	3·5	1·7	739	2259	3320	9·4	102	8·5
	Female: 10–12 years		142	3·6	1·2	675	1969	3560	8·7	89	8·0
	Female: 13–14 years		137	4·1	1·8	704	1867	2800	8·5	87	8·5
	Male: 7–9 years		164	3·7	2·1	876	2270	3520	9·7	111	8·8
	Male: 10–12 years		169	4·0	1·5	753	2215	3800	10·5	103	9·4
	Male: 13–14 years		143	3·9	1·4	649	2211	3540	10·3	94	9·4
Belgium	Belgian Food Consumption Survey 2014–2015	2014–2015									
	Female: 3–5 years		157	3·5	3·2	667		1511	6·4	114	
	Female: 6–9 years		174	3·6	3·2	672		1765	7·2	115	
	Female: 10–13 years		181	3·6	3·3	678		1905	7·7	116	
	Female: 14–17 years		185	3·6	3·3	684		1983	8·0	118	
	Male: 3–5 years		170	4·3	3·4	715		1555	7·2	111	
	Male: 6–9 years		189	4·4	3·4	744		2018	8·1	124	
	Male: 10–13 years		204	4·5	3·5	774		2318	9·4	136	
	Male: 14–17 years		214	4·6	3·6	799		2499	10·1	146	
Bulgaria	National Survey on Nutrition of Infants and Children Under 5 and Family Child Rearing 2007	2007									
	Female: 1 year		104	2·1	3·4	536		1400	5·0		5·2
	Female: 2 years		104	2·1	3·4	536		1400	5·0		5·2
	Female: 3 years		129	2·5	0·8	547		1873	6·3		6·5
	Female: 4 years		129	2·5	0·8	547		1873	6·3		6·5
	Male: 1 year		104	2·1	3·4	536		1400	5·0		5·2
	Male: 2 years		104	2·1	3·4	536		1400	5·0		5·2
	Male: 3 years		129	2·5	0·8	547		1873	6·3		6·5
	Male: 4 years		129	2·5	0·8	547		1873	6·3		6·5
Cyprus	A study of the dietary intake of Cypriot children and adolescents aged 6–18 years	2009–2010									
	Female: 6–8·9 years					930	2311	2283	10·9		
	Female: 9–13·9 years					876	2166	2289	10·7		
	Female: 14–18·9 years					859	2158	2294	10·4		
	Male: 6–8·9 years					957	2337	2331	11·4		
	Male: 9–13·9 years					929	2364	2515	11·5		
	Male: 14–18·9 years					1028	2515	2924	13·0		
Denmark	Danish Dietary Habits 2011–2013	2011–2013									
	Female: 4–9 years		270	5·1	2·5	966	2500	2800	8·4	210	8·9
	Female: 10–17 years		254	4·3	2·4	910	2500	3000	8·3	213	9·1
	Male: 4–9 years		289	5·6	2·8	1052	2700	3100	9·4	233	9·8
	Male: 10–17 years		307	6·0	3·1	1183	3100	3900	10·8	249	12·4
Estonia	National Dietary Survey	2014–2015									
	Female: 2–5 years		134	3·7	2·0	640	2300	1056	7·6	103	6·3
	Female: 6–9 years		155	3·9	2·1	724	2700	1299	9·0	116	7·3
	Female: 10–13 years		149	4·2	1·8	715	2600	1467	9·5	116	7·0
	Female: 14–17 years		162	4·5	2·5	630	2700	1434	9·6	104	7·4
	Male: 2–5 years		135	3·6	1·9	664	2500	1171	8·0	107	6·8
	Male: 6–9 years		175	6·4	3·1	861	3000	1549	11·0	148	8·9
	Male: 10–13 years		182	5·5	3·3	871	3100	1990	11·8	144	9·5
	Male: 14–17 years		201	5·4	3·2	907	3800	2196	13·2	157	11·0
France	INCA3	2014–2015									
	Female: 0–10 years		228	3·5	6·4	801	2020	1691	7·7	110	6·6
	Female: 11–17 years		270	3·9	2·8	859	2538	2352	8·9	122	7·7
	Male: 0–10 years		243	3·7	5·5	857	2224	1860	8·7	121	7·0
	Male: 11–17 years		300	5·0	3·0	996	2814	2832	10·7	146	9·6
Germany	German National Nutrition Survey II	2005–2007									
	Female: 14–18 years		340	4·0	2·0	1023	3011	2471	12·1	171	9·3
	Male: 14–18 years		410	6·3	2·7	1277	3655	3535	15·6	231	12·7
Ireland	National Pre-school Nutrition Survey	2010–2011									
	1 year		159	4·1	4·2	840	1716	918	7·0	182	5·4
	2 years		180	4·2	3·4	786	1724	1186	7·6	162	5·4
	3 years		188	3·8	3·0	718	1732	1250	7·2	139	5·2
	4 years		189	4·0	2·8	748	1830	1421	7·8	142	5·5
Ireland	National Children’s Food Survey	2003–2004									
	Female: 5–12 years		207	4·2	2·3	808			8·5		6·2
	Female: 5–8 years		204	4·3	2·2	815			8·4		6·0
	Female: 9–12 years		210	4·1	2·3	801			8·7		6·4
	Male: 5–12 years		243	4·7	2·2	918			10·3		7·1
	Male: 5–8 years		224	4·3	2·3	869			9·3		6·4
	Male: 9–12 years		261	5·0	2·2	965			11·2		7·6
Ireland	National Teens’ Food Survey	2005–2006									
	Female: 13–17 years		230	4·2	2·3	738			10·7		7·2
	Female: 13–14 years		221	4·1	2·3	725			12·4		7·0
	Female: 15–17 years		236	4·2	2·4	748			9·4		7·2
	Male: 13–17 years		320	6·0	3·0	1070			14·1		10·2
	Male: 13–14 years		396	6·0	2·8	1004			12·3		10·0
	Male: 15–17 years		338	6·1	3·1	1118			15·5		10·3
Italy	Third Italian National Food Consumption Survey INRAN-SCAI	2005–2006									
	All: 0–2·9			2·6	1·8	664	1471		5·4		5·6
	All: 3–9·9			5·7	2·0	749	2441		9·4		9·9
	Female: 10–17·9			6·5	2·4	770	2737		10·6		10·9
	Male: 10–17·9			6·9	2·6	892	3123		12·2		13·3
Latvia	Latvian National Food Consumption Survey 2007–2009	2007–2009									
	Female: 7–16 years							2000			
	Male: 7–16 years							2840			
Netherlands	Dutch National Food Consumption Survey – young children (DNFCS) 2008	2008									
	Female: 2–3 years		104	2·6	1·8	734			6·1		5·0
	Female: 4–6 years		107	2·5	1·9	748			6·7		5·2
	Male: 2–3 years		107	2·6	1·8	788			6·1		5·2
	Male: 4–6 years		119	2·9	2·2	854			7·1		5·9
Netherlands	Dutch National Food Consumption Survey (DNFCS) 2007–2010	2007–2010									
	Female: 7–8 years		164	3·3	2·3	817	2357		7·8		7·7
	Female: 9–13 years		179	3·3	2·4	892	2502	2257	8·2	141	8·2
	Female: 14–18 years		207	3·3	2·4	870	2622	2336	8·7	150	8·7
	Male: 7–8 years		161	3·0	2·3	878	2362		8·1		7·5
	Male: 9–13 years		202	3·7	2·8	943	2757	2544	9·2	164	9·1
	Male: 14–18 years		264	4·5	3·2	1093	3314	3064	10·6	193	11·1
Norway	UNGKOST 3	2015–2016									
	Female: 4 years		157	4·5	3·5	682	2000	1800	6·0		
	Female: 9 years		175	4·5	3·6	756	2200	2220	7·0		
	Female: 13 years		183	4·9	3·5	753	2300	2300	8·0		
	Male: 4 years		169	4·7	3·3	757	2200	1900	7·0		
	Male: 9 years		192	5·3	4·1	866	2500	2500	9·0		
	Male: 13 years		210	5·9	4·3	918	2700	2700	9·0		
Portugal	National Food and Physical Activity Survey (IAN-AF)	2015–2016									
	Female: <10 years		191·5	2·7	6·3	781	2504	1638	8·5		6·9
	Female: 10–17 years		222·4	4·5	3·5	757	2891	2731	10·7		9·7
	Male: <10 years		192·9	2·7	6·7	851	2539	1643	8·9		7·1
	Male: 10–17 years		251·8	5·1	4·3	922	3409	3255	12·1		12·1
Slovenia	Dietary Intake of Macro- and Micronutrients in Slovenian Adolescents	2012									
	Female: 15–16 years		276	5·9	4·0	1167	3770	4191	16·0	205	12·4
	Male: 15–16 years		255	6·7	4·0	1094	3494	4059	16·0	181	13·5
Spain	ANIBES	2013									
	Female: 9–12 years										
	Female: 13–17 years										
	Male: 9–12 years										
	Male: 13–17 years										
Turkey	Turkey Nutrition and Health Survey 2010 (TNHS)	2010									
	Female: 2–5 years		188	2·0	0·9	515	1593	971	6·6	44	5·6
	Female: 6–8 years		241	2·2	1·1	540	1925	1324	8·3	50	7·1
	Female: 9–11 years		277	2·3	0·9	549	2087	1587	9·6	52	7·9
	Female: 12–14 years		284	2·1	1·0	545	2049	1636	9·6	52	8·0
	Female: 15–18 years		284	2·3	0·8	562	2059	1560	9·7	54	8·0
	Male: 2–5 years		195	2·3	0·9	549	1675	1019	7·0	47	6·1
	Male: 6–8 years		243	2·9	1·6	553	1924	1453	8·7	51	7·5
	Male: 9–11 years		281	2·9	1·0	554	2039	1599	9·3	53	8·1
	Male: 12–14 years		344	3·4	1·2	603	2388	2009	11·5	56	9·5
	Male: 15–18 years		359	3·1	1·1	697	2430	2428	12·1	68	10·5
UK	National Diet and Nutrition Survey (NDNS) Years 1–4	2008–2012									
	Children: 1·5–3 years		150	3·9	1·9	773	1796	1307	6·3	142	5·2
	Female: 4–10 years		188	3·7	1·9	783	2084	1782	8·4	130	6·2
	Female: 11–18 years		186	3·5	1·9	670	2065	2600	8·4	109	6·3
	Male: 4–10 years		201	4·0	2·0	823	2211	1902	9·0	141	6·6
	Male: 11–18 years		233	4·7	2·4	889	2536	2960	10·7	141	8·3
